# A linguistic comparison between human- and AI-generated content

**DOI:** 10.1016/j.isci.2026.114976

**Published:** 2026-02-12

**Authors:** Flávia A. Rodrigues, Niclas F. Sturm, Flávio L. Pinheiro

**Affiliations:** 1 NOVA Information Management School (NOVA IMS), Universidade Nova de Lisboa, Lisboa, Portugal

**Keywords:** artificial intelligence, social sciences, linguistics

## Abstract

This study explores the linguistic differences between AI-generated content and human-written texts, particularly in Portuguese. We created two datasets: one with factual and false human-written texts, and another with texts generated by advanced, large language models (LLMs; GPT-4o, Mistral Large, and Llama 3.3 70B), using various prompts. Using tools like linguistic inquiry and word count (LIWC) and sparse additive generative model (SAGE), we identified distinctive traits: AI-generated text tends to be more formal, structured, positive, and motivational, while human texts vary more in length, exhibit negative emotions, and often use personal references. Additionally, a misinformation detection model performed well on human texts (93% accuracy) but struggled with LLM outputs (75% accuracy). This highlights the unique linguistic patterns of AI-generated misinformation and underscores the need for better detection methods to tackle misleading content in Portuguese.

## Highlights

• We compare the linguistic characteristics of content authored by humans and LLMs
• This study focuses on content in Portuguese and contrasts true and false information
• We show that AI-generated misinformation is more formal, positive, and structured
• Such characteristics affect the performance of misinformation detection models

## Graphical abstract

**Figure:**
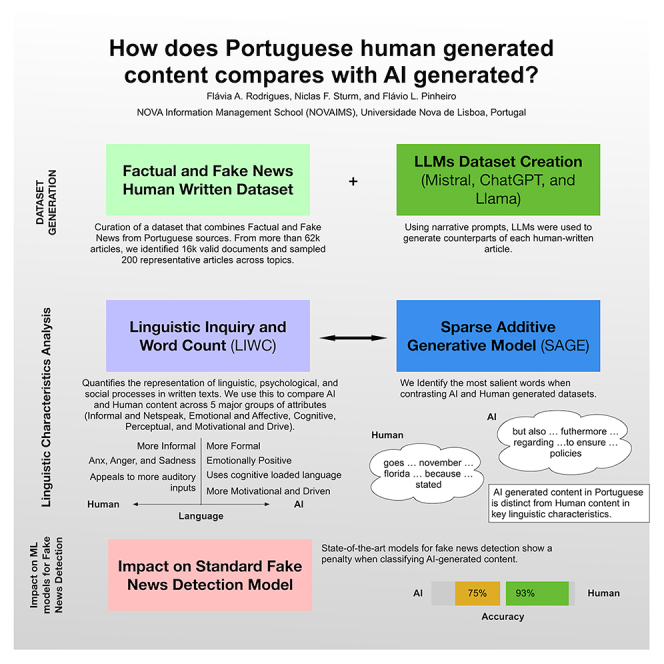

## Introduction

Recent developments in AI have significantly increased the scale and complexity of the content shared across social media platforms.^1^ In particular, misinformation, disinformation, and other forms of deceptive content are now widely disseminated through online platforms, with few barriers to their creation and distribution. Individuals can publish and share false claims or content without verifying their sources or disclosing their identities. Global institutions and actors have raised significant concerns regarding the impact of misleading content on democratic processes,^2^^,^^3^ public health,^4^^,^^5^^,^^6^ economy,^7^ and social cohesion.^8^

In that context, large language models (LLMs) represent a technological turning point in content generation. Indeed, LLMs can generate highly persuasive text that closely mimics human writing, offering the potential for enhancing productivity and creativity.^9^^,^^10^ Still, they present new challenges, particularly when employed on a large scale and deliberately to produce misleading content. The realistic nature of AI-generated content makes it harder for conventional approaches—involving both humans and those supported by automated systems—to distinguish whether the content was created by a human or a machine.^11^^,^^12^

Although the topic of fake news has been extensively studied,^13^ most research has concentrated on the psychological and sociological factors driving the spread of misinformation^7^^,^^14^^,^^15^ or evaluating detection methods for human-generated content.^16^^,^^17^^,^^18^^,^^19^

Fewer studies have compared the linguistic features of misinformation generated by LLMs and humans^12^^,^^20^ and those that do tend to focus on English, limited domains, or specific model types. To the best of the authors’ knowledge, no study has yet examined this phenomenon in Portuguese, nor with a combined focus on both factual and fake news generated by multiple LLMs across diverse topics. This gap in the literature is particularly relevant as LLMs become more integrated into communication systems; it is essential to understand what distinguishes human-generated content from machine-generated content across the diversity of languages that comprise our online communities.

Here, we examine how emotional, cognitive, and stylistic linguistic features can help determine whether a news article was authored by a human or generated by an AI model. The analysis focuses on Portuguese language news articles, both factual and false, across various domains such as politics, sports, health, and finance, which we compare with content generated by three state-of-the-art LLMs (GPT-4o, Mistral Large, and Llama 3.3 70B).

We show that LLM-generated texts exhibit distinct linguistic patterns compared to human-written content. AI-generated texts tend to be more formal, structured, emotionally positive, and motivational. They prefer visual descriptions and show a subtler use of cognitive expressions. In contrast, human-authored content is characterized by greater variability in length, negative emotional intensity such as anger and sadness, and a more informal style and use of specific temporal and personal references. We found that these tendencies generalize over both factual and false news, pointing to more general differences in expression between human-written and AI-generated texts. Despite subtle stylistic differences between models, Llama shows greater emotional expressiveness, and ChatGPT presents a more neutral and polished style, with their general linguistic tendencies remaining aligned.

Cross-linguistic analysis reveals similar trends in English, although Portuguese LLM outputs contain fewer offensive terms, display fewer cognitive indicators, and tend to favor visual over auditory references. These differences suggest that AI-generated misinformation adapts to language-specific stylistic norms, which may be shaped by cultural expectations within Portuguese-language journalism—such as a preference for a more descriptive, less confrontational tone. While some of these patterns may be partially attributable to the linguistic structures common to Romance languages (e.g., higher inflectional complexity and subject-drop phenomena), the relative absence of emotionally charged or cognitively heavy expressions in Portuguese LLM outputs appears to reflect sociocultural norms specific to Portuguese-speaking media environments. Further cross-linguistic research across other Romance languages (e.g., Spanish, Italian, and French) is required to determine the extent to which these patterns are generalizable.

Furthermore, an advanced misinformation detection model demonstrated high accuracy (93%) for human-generated texts but significantly lower performance (75%) in AI-generated content. By focusing on the Portuguese context and comparing misinformation generated by different LLMs, this research contributes to a better understanding of how LLMs are reshaping the landscape of misinformation. Furthermore, it highlights that detection models can vary in performance depending on the type of content, emphasizing the need to choose suitable approaches to identify deceptive information accurately.

### Literature review

Identifying the veracity of news is a significant challenge for humans.^15^^,^^21^ A common belief is that confirmation bias and politically motivated reasoning contribute to people’s difficulty in distinguishing between factual and false content.^22^^,^^23^^,^^24^ In other words, people are more likely to accept information as accurate if it supports their existing views and dismiss information as false if it does not. However, evidence shows that people are more likely to believe factual news that challenges their political opinions than news that supports them but is false.^14^^,^^25^ Indeed, careful reasoning, familiarity, motivation, and engagement with content are key mechanisms for people to identify false content,^15^ which are moderated/influenced by traits such as education, analytical thinking, political orientation, age, and confidence.^26^^,^^27^

Fake news and misleading content are often characterized by high sensationalism, clickbait, deceptive content, and partisan bias.^28^ Additionally, fake news tends to be more novel than factual news, often containing more unexpected and original information compared to established reports. As such, fake news usually causes strong emotions such as fear, disgust, and surprise, as reflected in people’s reactions on social media.^29^ LLMs have demonstrated the ability to generate content that closely resembles human writing^12^ and, indeed, is capable of replicating crucial aspects of human language processing,^30^ blurring the line differentiating between human- and AI-generated content.^31^^,^^32^^,^^33^ Indeed, in some cases, not only do people struggle to distinguish the source, but AI-generated content can be perceived as more credible.^11^^,^^34^

LLMs have introduced a new field of play concerning the production of false content.^33^ That not only means, for instance, an increased ability to generate false news, instances of fraudulent academic contributions, and concerns related to social security,^35^ but also that the barriers for individuals, regardless of their intentions or expertise to produce such content, have drastically reduced. Hence, the rapid progress in AI-driven generative capabilities is expected to lead to the widespread dissemination of false information on a massive scale.^36^ Hence, it is imperative to develop our capabilities to detect content generated by LLMs to combat and mitigate the misuse of such models.^33^

A combination of machine learning, natural language, and network-based methods is commonly used to identify and flag problematic content.^37^^,^^38^^,^^39^ Indeed, AI can help identify fake news or misinformation. Still, its effectiveness is limited, especially when dealing with AI-generated content, which often meets criteria for credibility, transparency, and comprehensiveness, yet continue to mislead. This makes it harder for detection models to identify such content accurately and raises concerns about the relevance and reliability of current information assessment guidelines.^12^ Moreover, existing detection models were primarily designed to address traditional forms of misinformation, which limits their ability to generalize to newer, AI-generated forms. As a result, studies show that these models experience significant performance drops when confronted with synthetic content produced by LLMs.^12^^,^^40^^,^^41^

AI-generated misinformation related to COVID-19 and produced by GPT-3 displays key linguistic differences compared to humans: it tends to be more emotional and use more cognitive expressions, which may enhance its perceived credibility.^12^ Interestingly, AI-generated texts were more formal, whereas human-authored content appeared more spontaneous and natural. Consequently, existing models for fake news identification performed significantly worse on AI-generated content, which aligns with the past reports of models suffering significant performance penalties when facing AI-generated content.^42^ Similarly, Muñoz-Ortiz et al.^20^ compared human-authored and AI-generated texts, analyzing outputs from multiple LLMs—Llama (7B, 13B, 30B, and 65B), Falcon 7B, and Mistral 7B—to show that human texts exhibit a greater variety in sentence length, vocabulary, grammatical patterns, and shorter sentence components and more efficient dependency distances. Human-written texts also express stronger negative emotions (e.g., fear and disgust) and less joy than LLM-generated content. In contrast, LLMs used more numbers, symbols, auxiliary verbs, and pronouns. Biases such as sexism^43^ present in human texts are often mirrored and sometimes amplified in LLM outputs.^44^ However, it is crucial to expand into different languages, beyond English, and modern domains to gain a broader understanding of the differences between AI-generated content and human-authored content.

Cai et al.^30^ evaluated ChatGPT (GPT-3.5) and Vicuna by using 12 psycholinguistic tests, comparing their performance to human participants and showing that LLMs, especially ChatGPT, approximated human performance in several tasks, reinforcing the idea that such models capture key elements of human language processing. Xu et al.^45^ explored how GPT-3.5 and GPT-4 represent concepts, focusing on their alignment with human assessments across five psycholinguistic dimensions, and found that both models aligned well with human ratings in abstract areas like emotion and salience but struggled with sensory and motor domains. Giorgi et al.^46^ studied true and false hotel reviews written by humans and the false ones generated by ChatGPT-3.5. They analyzed traits like age, gender, and personality, showing that human texts displayed more variation in psychological markers. Rosenfeld et al.^34^ explored linguistic differences between GPT-3.5, GPT-4, and Bard. Using basic classification models, they could attribute texts to their LLM source with 88% accuracy based on linguistic features such as length, complexity, and density. These results suggest that human psychological features help distinguish human writing from AI writing. Explicitly defined linguistic features are being increasingly applied to the detection of AI-generated writing, often focusing on aspects such as grammatical and situational features, as well as text complexity.^47^^,^^48^^,^^49^^,^^50^^,^^51^

Overall, past work has shown that while advanced AI models can generate content that is hard to differentiate from human writing, linguistic methods emerge as a promising approach to provide transparent insights to distill the differences of content producers, making them also a reliable pathway to distinguishing between AI-generated and human-authored content.^20^^,^^34^^,^^52^

## Results

### Descriptive linguistics analysis

We proceed to a descriptive analysis of the text corpora. A descriptive summary of topics covered by the human-authored dataset, which comprises both factual and fake news, and which shows notable breadth in thematic coverage, can be consulted in Table 1. Following the narrative-lead approach of text generation, which we illustrate in Table 2, we can now directly compare these two corpora, human-generated and AI-generated.

**Table 1:** Top 5 keywords and corresponding frequencies for each LDA estimated topic in the true and fake news datasets

Topic	Factual news		Fake news	
	Top keywords	Frequency	Top keywords	Frequency
1	government, measure, euro	7.02%	year, euro, bank	9.52%
2	case, court, trial	4.42%	woman, life, home	13.29%
3	government, power, party	11.54%	Portugal, country, power	7.51%
4	Lisbon, city, place	6.44%	water, power, beach, home	5.13%
5	year, victim, authority	6.11%	km_radar, Lisbon, road	3.16%
6	NATO, Turkey, fire	2.14%	power, person, good	9.21%
7	Ukraine, Russian, Russia	6.93%	car, power, vehicle	4.39%
8	power, health, year	13.98%	company, Portugal, state	7.16%
9	power, year, person	8.43%	court, defendant, mass	1.90%
10	country, European, EU	6.98%	system, model, technology	2.22%
11	power, United States, world	4.84%	government, deputy, euro	5.18%
12	company, euro, year	9.57%	doctor, hospital, day, power	4.88%
13	Portuguese, Portugal, power	7.04%	world, person, video, year	8.71%
14	game, team, year	4.54%	Portuguese, player, name	3.58%
15	–	–	child, father, year	8.83%
16	–	–	power, body, day	5.35%

For the true news dataset, although the model with k=18 topics achieved the highest coherence score,^57^ the model with k=14 offered better separation and interoperability of topics. Likewise, despite not maximizing coherence, we chose k=16 topics for the fake news dataset to ensure closer alignment with the number of topics in the real news dataset and to promote greater textual diversity across clusters. We used the gensim implementation of LDA in Python.^58^

**Table 2:** Narrative prompt variables and examples

Variable	Description	Prompt Example
Story	the content of the narrative, including the main events, actions, and entities described in the article.	old recipe that is being promoted as a natural cure for cancer
Type	the format of the text, which, in this study, was always news.	a news item
Purpose	the communicative intent of the text (e.g., report, instruction, and commentary).	reporting to give instructions
Evidence	citations or supporting information used to validate the news, when applicable.	according to the World Health Organization (WHO)

**Figure 1:**
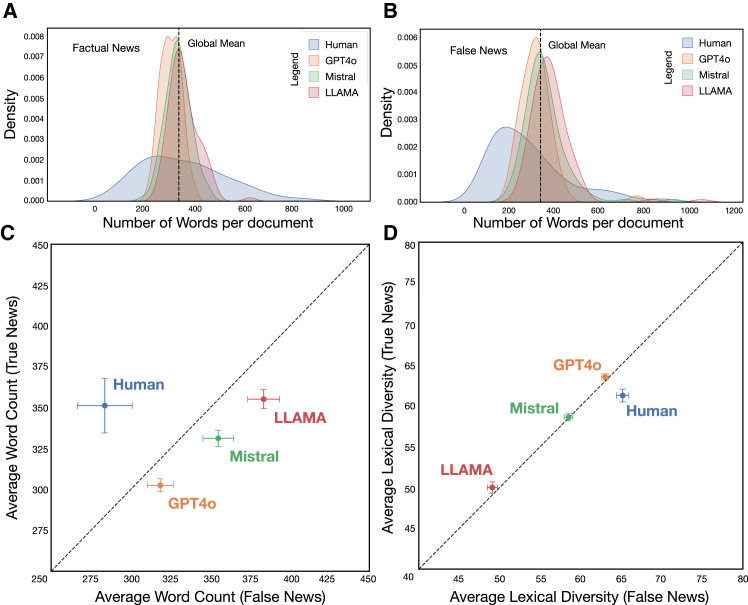
Descriptive comparison between the documents produced by humans and LLMs (A and B) Comparison of the distribution between factual (A) and false (B) news in terms of word count (length) of the articles. (C and D) Comparison of both factual and false news by either source, humans or LLMs, in terms of average document size (C) and average lexical diversity (D). Word count (factual/fake news) and lexical diversity (factual/fake news) are represented as the mean word count/lexical diversity ± standard deviation. LLMs, large language models.

Figure 1 compares the articles produced by humans and AI models in terms of document length (i.e., number of words) and lexical diversity. Figures 1A and 1B reveal that human-authored texts exhibit greater variability in length, characterized by a broader and flatter distribution. In contrast, AI-generated texts, particularly those from GPT-4o and Mistral, are more uniform, clustering closely around the average. This suggests that LLMs tend to produce more standardized outputs, whereas human writing is inherently more variable. More importantly, LLMs appear to be relatively consistent in terms of document length, regardless of the content’s truthfulness.

When comparing their average length, in the case of factual news (Figure 1C, Y axis), Llama generates the longest texts on average, slightly surpassing even those produced by humans. Conversely, GPT-4o tends to create shorter and more uniform outputs. For fake news (Figure 1C and X-axis), all LLMs produce longer texts than humans, with Llama again being prominent. As a consequence, AI models, particularly Llama, tend to overproduce in deceptive contexts, potentially rendering fake content more elaborate or convincing.

Regarding lexical diversity (Figure 1D), GPT-4o exhibits the highest variety in real news, surpassing human output, whereas Llama consistently employs a more restricted vocabulary. In the case of fake news, humans demonstrate the richest vocabulary, reinforcing the notion that AI-generated texts, especially those from Llama, are more repetitive.

### LIWC analysis

Linguistic inquiry and word count (LIWC) analysis, as shown in Table 3, revealed systematic and statistically significant linguistic differences between human- and AI-generated texts across all five dimensions. Compared to humans, all LLMs consistently produced content with a more formal and standardized tone, using significantly fewer informal and netspeak expressions. This was especially evident in fake news, where humans tended to adopt a more casual and spontaneous style, often resembling everyday speech or discourse from social media. In contrast, LLMs maintained a polished tone, which may reflect how these models aim to sound credible and avoid informal or potentially risky language, even when generating deceptive content.

**Table 3:** Linguistic differences between human- and AI-generated texts across all five LIWC categories

LIWC Category	Factual News			Fake News		
	ChatGPT	Mistral	Llama	ChatGPT	Mistral	Llama
**Informal and Netspeak attributes**						

informal (informal language)	**−54.42%∗∗**	**−55.99%∗∗**	**−72.22%∗∗∗**	**−63.56%∗∗∗**	**−53.61%∗∗∗**	**−64.16%∗∗∗**
netspeak (Netspeak)	−38.56%	−32.04%	**−69.76%∗**	**−83.13%∗∗∗**	**−81.64%∗∗∗**	**−83.81%∗∗**

**Emotional and Affective Attributes**						

affect (Affect)	**96.19%∗∗∗**	**99.75%∗∗∗**	**111.46%∗∗∗**	**42.24%∗∗**	**53.76%∗∗**	**67.17%∗∗∗**
posemo (Positive Emotions)	**114.19%∗∗∗**	**119.22%∗∗∗**	**132.96%∗∗∗**	**69.96%∗∗**	**76.79%∗∗**	**78.95%∗∗**
negemo (Negative Emotions)	40.08%	19.89%	44.37%	4.79%	9.37%	21.50%
anx (Anx)	57.31%	4.92%	19.90%	**−46.08%∗∗**	**−43.91%∗∗∗**	**−39.39%∗∗**
anger (Anger)	**−15.33%∗**	**−22.93%∗**	−19.42%	**−33.28%∗∗**	**−51.12%∗∗∗**	**−40.05%∗∗**
sad (Sad)	**−30.60%∗**	−23.48%	**−39.45%∗∗**	**−17.98%∗**	**−16.56%∗**	36.66%

**Cognitive Attributes**						

cogproc (Cognitive Processes)	**50.72%∗∗∗**	**54.37%∗∗∗**	**54.36%∗∗∗**	21.94%	26.40%	33.36%
insight (Insight)	49.09%	22.21%	42.94%	**57.15%∗∗**	52.07%	49.34%
cause (Causal)	**52.23%∗∗∗**	**60.53%∗∗∗**	**67.14%∗∗∗**	**42.04%∗**	**38.01%∗∗**	**53.63%∗∗∗**
discrep (Discrepancies)	22.09%	20.90%	**43.22%∗∗**	**−14.80%∗∗∗**	**−7.39%∗∗**	5.77%
tentat (Tentative)	59.67%	47.32%	**72.55%∗**	**−13.81%∗∗**	11.96%	26.50%
certain (Certainty)	**152.49%∗∗∗**	**142.86%∗∗∗**	**111.00%∗∗∗**	21.43%	39.11%	23.53%
differ (Differentiation)	34.21%	27.18%	32.62%	**4.44%∗**	11.33%	20.50%

**Perceptual Attributes**						

percept (Perceptual Processes)	34.06%	43.03%	52.44%	−6.24%	2.54%	17.75%
see (See)	46.12%	44.63%	22.60%	20.41%	37.52%	6.00%
hear (Hear)	**−49.78%∗∗∗**	**−34.82%∗∗∗**	**−19.35%∗**	**−63.44%∗∗∗**	**−30.80%∗∗**	**−22.99%∗**
feel (Feel)	−11.39%	−4.51%	**7.86%∗**	**−25.15%∗∗**	**−33.22%∗∗∗**	**−14.72%∗∗**

**Motivational and Drive Attributes**						

drives (Drives)	**46.93%∗∗∗**	**42.24%∗∗∗**	**56.58%∗∗∗**	**36.81%∗∗∗**	**37.73%∗∗∗**	**44.94%∗∗∗**
affiliation (Affiliation)	**79.12%∗∗∗**	**42.42%∗**	28.28%	17.63%	19.63%	15.70%
achieve (Achievement)	**113.01%∗∗∗**	**104.02%∗∗∗**	**131.89%∗∗∗**	**68.23%∗∗∗**	**68.29%∗∗∗**	**64.89%∗∗∗**
power (Power)	17.59%	22.88%	**31.30%∗∗**	**49.55%∗∗**	**55.01%∗∗∗**	**53.22%∗∗∗**
reward (Reward)	**70.72%∗∗∗**	**80.15%∗∗∗**	**80.55%∗∗∗**	34.12%	**54.17%∗∗**	**57.28%∗∗∗**
risk (Risk)	**85.46%∗∗∗**	**77.78%∗∗∗**	**111.95%∗∗∗**	42.26%	**58.70%∗∗**	**81.74%∗∗∗**

Each cell shows the relative difference (Δ%) between each LLM (GPT-4o, Mistral, and Llama) and the human baseline, along with the significance levels obtained using the Wilcoxon signed-rank test. Category comparison across LLMs for factual and fake news. Positive values in bold indicate significantly higher usage by LLMs; negative values in bold indicate significantly lower usage. Significance levels after Benjamini-Hochberg correction for multiple comparisons are based on the Wilcoxon signed-rank test: p<0.05(^∗^), p<0.01((^∗∗^), p<0.001 ((^∗∗∗^).

AI-generated texts also showed a marked preference for positive and motivational language, possibly to make the content more engaging or relatable to readers. In contrast, negative emotions—especially anger and sadness—were consistently reduced. This suggests a tendency for LLMs to avoid sensitive or potentially harmful content, regardless of whether the text was factual or deceptive. This behavior is likely the result of alignment mechanisms that steer models away from producing upsetting or inappropriate material. The trend was even more pronounced in fake news, where humans used more expressions of negative affect to provoke stronger emotional reactions or to make messages more dramatic and persuasive.

Additionally, cognitive language also increased in AI-generated texts, particularly in real news, where higher usage of certainty and causal expressions contributed to a more structured and authoritative style. In fake news, however, LLMs tended to adopt a more assertive tone, showing fewer markers of doubt or discrepancies than human writers.

Regarding perceptual language, there was a consistent emphasis on visual descriptions over auditory or other sensory terms, reflecting the tendency of models to be trained predominantly on descriptive and visually oriented information, rather than on experiences grounded in embodied human perception.

**Figure 2:**
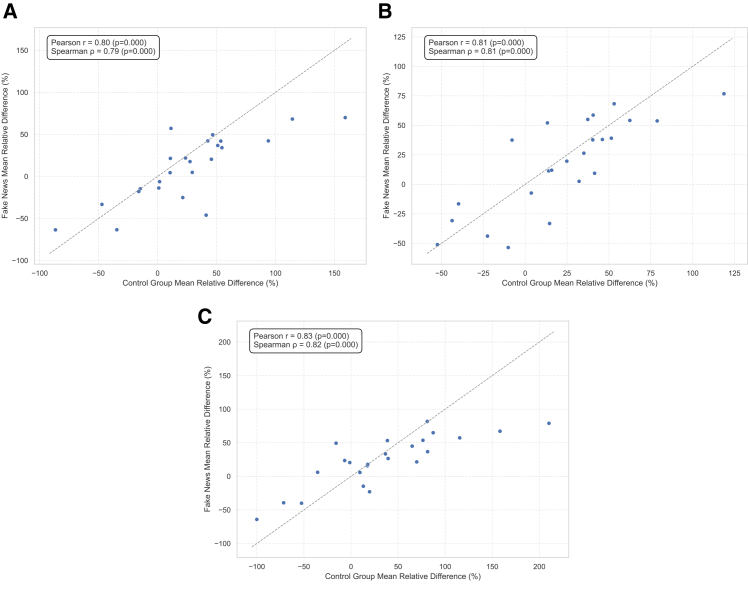
LIWC category correlation between control group and fake news (A) ChatGPT. (B) Mistral. (C) LlaMA. Each scatterplot shows the correlation analysis (Pearson’s and Spearman’s) to assess whether linguistic patterns in fake news articles align with those in pre-AI human-generated content (the control group). LIWC, linguistic inquiry and word count.

While all three LLMs followed similar patterns, Llama generally exhibited more substantial increases in affective and motivational attributes, suggesting a more emotionally expressive style. Mistral maintained a balanced profile across categories, and GPT-4o showed a more neutral and polished output with comparatively low emotional intensity. We conducted a robustness test comparing LIWC category correlations between the three LLMs with pre-LLM human-generated content, the results of which are summarized in Figure 2. Those results show strong concordance with the results obtained here as well.

These findings align with those of previous work focusing on fake news in English^12^ but also reveal specific nuances in the Portuguese dataset, such as the lower prevalence of negative emotional expressions (e.g., sadness, anger, and anxiety) and a more substantial reliance on visual descriptions in LLM-generated texts. This may reflect cultural- and language-specific norms as well as the evolution of LLM architectures and alignment techniques. Cognitive markers were present but less pronounced than those in English, suggesting a subtler use of reasoning cues.

### SAGE analysis

Sparse additive generative model (SAGE) analysis provided an additional layer of linguistic differentiation by identifying the most salient keywords that distinguish AI-generated texts from those authored by humans. By examining uni-, bi-, and tri-grams, the model captured not only isolated word choices but also multi-word expressions. Table 4 compares the SAGE analysis of human-written news with the GPT-4o-, Mistral- and Llama-generated content; the original results in Portuguese are presented in Table S3.

**Table 4:** Salient keywords distinguishing human-authored and AI-generated texts identified by SAGE

Model	News type	LLM keywords	Human keywords
ChatGPT	Factual News	**but also** (−3.89); as it progresses (−3.64); meanwhile (−3.33); in the region (−3.24); **furthermore** (−3.24); not only (−3.24); incident (−3.24); may (−3.14); reinforce (−3.14); **regarding** (−3.03)	**goes** (3.89); face (3.46); count (3.40); **november** (3.40); 8:00 (3.33); **stated** (3.26); july (3.26); **president of the republic** (3.19); **florida** (3.19); **euros m2** (3.19)
ChatGPT	False news	**importance** (−4.31); **to ensure** (−3.79); **but also** (−3.48); **policies** (−3.05); management (−3.05); not only (−2.96); of the country (−2.96); through (−2.96); this sign (−2.86); **including** (−2.75)	**so** (4.14); then (3.51); **their** (3.43); **are people** (3.35); km (3.35); **goes** (3.32); **because** (3.28); salary (3.26); person (3.26); blood (3.16)
Mistral	Factual news	it’s crucial (−3.75); ministry of (−3.47); transparency (−3.39); real estate market (−3.30); **but also** (−3.21); sweden’s accession (−3.21); a moment (−3.21); to monitor (−3.21); concerns (−3.21); international community (−3.11)	statement (3.60); **november** (3.42); account (3.42); **because** (3.36); **stated** (3.29); **florida** (3.21); **euros m2** (3.21); third (3.21); secretary-general (3.13); sweden nato (3.04)
Mistral	False news	**to ensure** (−4.42); **importance** (−4.28); **including** (−3.42); to avoid (−3.22); commented (−3.06); **but also** (−2.98); not yet (−2.98); has generated (−2.89); if you (−2.89); **policies** (−2.79)	**so** (4.18); **goes** (4.05); people (3.55); go (3.55); deposit (3.55); **their** (3.47); **are people** (3.39); **because** (3.32); month (3.30); as (3.25)
Llama	Factual news	**furthermore** (−4.28); marcelo rebelo (−3.70); **regarding** (−3.55); a sign (−3.55); patients (−3.55); vaccination against (−3.55); in the coming days (−3.47); it’s a step (−3.47); an experience (−3.47); **but also** (−3.39)	marcelo rebelo de (3.65); **stated** (3.55); thursday (3.49); group (3.43); donald trump (3.37); **november** (3.37); **president of the republic** (3.16); **euros m2** (3.13); today (3.07); wednesday (3.07)
Llama	False news	a reminder (−4.09); **importance** (−3.84); it’s fundamental (−3.59); **including** (−3.59); the consumers (−3.53); promote (−3.47); of the accident (−3.33); may have (−3.26); of the party (−3.10); **but also** (−3.01)	**so** (4.12); **their** (3.42); **are people** (3.34); km (3.34); almost (3.34); in this (3.34); thousand (3.15); ave (3.15); happen (3.15); radar (3.15)

Original versions in Portuguese can be found in Table S4. In bold, keywords occurring in at least two distinct LLMs are highlighted. Similarly, keywords from human content that differ from those of two distinct LLMs a highlighted in bold.

Human content, especially factual news, frequently included time-specific expressions such as “novembro” (November) and “terça-feira” (Tuesday), reflecting a referential communication style aligned with journalistic norms. However, false news authored by humans often relied on more informal and everyday phrases like “por isso” (therefore), “vai” (go), “os seus” (their), and “são pessoas” (are people), creating a tone that appears more spontaneous and socially grounded. Conversely, AI-generated texts consistently favored formal and cognitive language such as “mas também” (but also), “além disso” (furthermore), “em relação” (regarding), “importância” (importance), and “é fundamental” (is fundamental). This pattern supports the LIWC findings of increased cognitive and drive-related language, as well as a reduced presence of informal markers, in LLM outputs. This indicates that AI models produce structured and polished language that sounds more authoritative or instructive, even when generating false news. In contrast, human-authored texts exhibit a more natural and conversational style.

Similar patterns emerged in the control group, which consisted of texts verified as being written by a human before the widespread use of generative AI. The SAGE analysis confirmed that these false news texts also contained socially grounded and conversational expressions such as “ficar” (stay), “doentes” (sick), “casa” (home), “do Sporting” (from Sporting), and “estado” (state). This convergence between the primary dataset and the control group further reinforces the assumption that humans indeed authored the fake news articles analyzed in this study.

Although all language models displayed a tendency toward formal expressions, some subtle distinctions emerged. For example, ChatGPT often favored abstract and formal terms such as “importância” (importance) and connectors like “mas também” (but also). At the same time, Mistral outputs included a slightly broader range of expressions related to formal instructions (“é essencial” [is essential], “para garantir” [to ensure]). Llama, in turn, frequently produced phrases conveying structured elaboration and emphasis (“além disso” [furthermore], “um lembrete” [a reminder]).

Compared to English-language findings,^12^ the Portuguese SAGE results revealed broadly similar patterns; however, temporal markers were less prominent in Portuguese fake news and appeared more clearly in real news, whereas in the English dataset, they were salient in fake news.

### Fake news detection models

**Figure 3:**
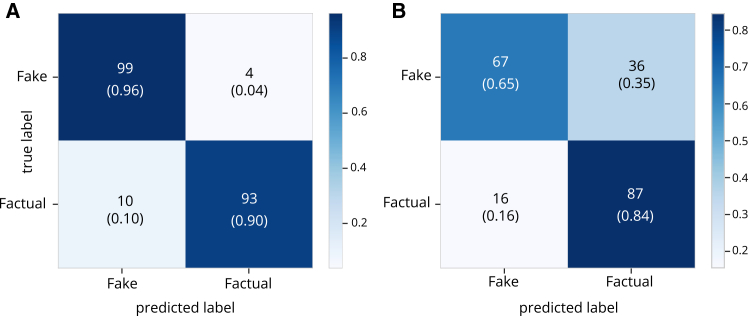
Confusion matrix for the XGBoost model (A) The results for human-authored news. (B) The results for LLM-generated news.

Using the fake news detection model proposed by Afonso and Rosas,^53^ we show that the model achieves the highest performance on human-generated content (see Figure 3A), with an accuracy of 93% and an average F1-score of 0.93, and a balanced classification between fake and real texts. Indeed, the model can correctly identify 99 out of the 103 fake news articles and 93 out of the 103 factual news articles, resulting in a low false-negative rate.

However, model performance dropped considerably when applied to LLM-generated content. The accuracy decreased to 0.75, and the macro-average F1-score decreased accordingly. The model encountered particular difficulties in detecting fake news, misclassifying 36 out of 103 fake news items (see Figure 3B and Table 5). Although it maintained reasonable performance on real news, the decline in both categories suggests that LLM-generated content presents challenges to conventional detection models.

**Table 5:** Performance metrics for the XGBoost classifier on human-written and LLM-Generated news

Dataset	Precision	Recall	F1-score	Accuracy
Human-written news	0.93	0.93	0.93	0.93
LLM-generated news	0.76	0.75	0.75	0.75

Precision, recall, and F1-score are macro-averaged.

A chi-squared test was conducted to test whether these performance differences are statistically significant. The results revealed a highly significant difference ($\chi^{2}=22.66$, p<0.001), confirming that the detection model performs differently depending on whether the input was written by a human or generated by an AI model. These results are consistent with findings from previous research in English.^12^ Traditional detection models trained on human-authored texts may not generalize to AI-generated content. This highlights the need for updated detection strategies that account for the unique linguistic and structural features of LLM outputs. Future efforts should focus on retraining detection models with diverse training data, including synthetic examples, and leveraging interpretable linguistic cues identified in this study.

## Discussion

In this study, we investigated the linguistic characteristics of factual and false news articles authored by humans and generated by three LLMs—GPT-4o, Mistral Large, and Llama 3.3 70B—in Portuguese. Integrating descriptive metrics with analyses using LIWC and SAGE, we identified consistent and statistically significant differences between LLM-generated and human-authored content. These differences remain robust over different LLM text-generation parameters, which we have summarized, including the reference value, in Table 6.

**Table 6:** LLM generation parameters—reference and variation

Description	Temperature	${Top}_{p}$	Descriptive Analysis	SAGE	LIWC
Reference	0.7	1	Figure 1	Table 2	Table 1
–	0.5	1	Figure S3	Table S6	Table S7
	0.7	0.5	Figure S4	Table S8	Table S9
	0.7	0.8	Figure S5	Table S10	Table S11
	0.9	1	Figure S6	Table S12	Table S13

The higher positivity, formality, and structural coherence of LLM-generated misinformation may inadvertently enhance its credibility and persuasive power, making it more difficult for both human readers and automated systems to detect deception. Prior studies have shown that formal tone and emotional neutrality are often associated with trustworthiness, which suggests that AI-generated fake news could bypass intuitive credibility filters. Furthermore, the subtle emotional cues and visual descriptiveness observed in LLM content may appeal to readers cognitively and emotionally, potentially facilitating wider dissemination, especially on social media platforms where attention and engagement are key drivers of virality.

A further contribution of this study is the evaluation of an existing fake news detection model trained on human-written texts. While the model performs well on human data, its performance declines significantly when applied to LLM-generated texts, particularly in detecting AI-generated fake news. These results underscore the concern that detection systems based on traditional linguistic cues may fail to identify misinformation created by modern generative models. This threat underscores the need for adaptive, transparent detection systems,^54^^,^^55^^,^^56^ including models trained on mixed-origin datasets and enriched with interpretable features. Ongoing monitoring of evolving LLM styles and adversarial tactics will be essential to maintain effective detection in dynamic information environments. Future work should explore incorporating AI-generated misinformation into detection models to increase the exposure to LLM-generated content and its linguistic idiosyncrasies. Furthermore, interpretable linguistic features, such as those employed in this article, could serve as additional fingerprints to strengthen detection robustness.

By uncovering the linguistic patterns of AI-generated misinformation in Portuguese and assessing their detectability, this study contributes to the broader effort to anticipate, understand, and mitigate the evolving risks of generative language models in digital information environments.

### Limitations of the study

Despite its contributions, this study has several limitations that open avenues for future research. While the analysis focused on Portuguese, an underrepresented language in misinformation research, the findings may not fully generalize to other linguistic or cultural contexts. Future work should replicate similar analyses in additional languages beyond English and Portuguese to assess the cross-linguistic consistency of AI-generated misinformation patterns. Another limitation relates to the assumption of human authorship in a real dataset. As generative tools become increasingly embedded in everyday writing, especially online, it becomes harder to ensure that all texts are genuinely written by a human. Future studies could address this issue by designing surveys that explicitly ask participants to produce both fake and real content, thereby ensuring controlled authorship and enabling more direct comparisons.

Moreover, the LLMs assessed in this study reflect a particular generation of architectures whose capabilities are advancing at a remarkable pace. As these models evolve, it will be essential for future research to revisit and expand these analyses using newer and alternative systems to track how linguistic characteristics change over time systematically. From a methodological perspective, text collection and prompt design were time-intensive tasks. Expanding this work to larger datasets, with more automated or scalable pipelines, would improve robustness and generalizability. Additionally, while the fake news detection model evaluated here performed well on human-written content, it encountered difficulties with AI-generated misinformation. This performance disparity underscores the urgent need to retrain classifiers by using hybrid datasets that incorporate synthetic texts and to integrate interpretable linguistic cues, such as those identified through this study.

## Resource availability

### Lead contact

Requests for further information and resources should be directed to and will be fulfilled by the lead contact, Flávio L. Pinheiro (fpinheiro@novaims.unl.pt).

### Materials availability

This study did not generate new materials.

### Data and code availability

• We have ensured that all the essential data necessary for replicating our results are included in the GitHub repository, which reports the generated texts and links to the published data from Rosas and Afonso (2024).
• Original code is provided in a GitHub repository.
• Additional information required to reanalyze the data reported in this paper is available from the lead contact upon request.

## Acknowledgments

This work was supported by national funds through 10.13039/501100001871FCT, under the projects UID/04152/2025: Centro de Investigação em Gestão de Informação (MagIC)/NOVA IMS (https://doi.org/10.54499/UID/04152/2025; 2025-01-01/2028-12-31) and UID/PRR/04152/2025 (https://doi.org/10.54499/UID/PRR/04152/2025; 2025-01-01/2026-06-30). F.L.P. and N.F.S. also acknowledge financial support of FCT under the project Know-Net-Compet (https://doi.org/10.54499/2024.07378.IACDC).

## Author contributions

Conceptualization, F.A.R. and F.L.P.; methodology, F.A.R. and F.L.P.; investigation, F.A.R.; writing – original draft, F.A.R., F.L.P., and N.F.S.; writing – review & editing, N.F.S., F.A.R., and F.L.P.; supervision, F.L.P. and N.F.S.

## Declaration of interests

The authors declare no competing interests.

## STAR★Methods

### Key resources table

**Table:** 

REAGENT or RESOURCE	SOURCE	IDENTIFIER
**Deposited data**		

Fake News Dataset (PT)	Published Data	https://github.com/ro-afonso/fake-news-pt-eu.git

**Software and algorithms**		

Python	Python Software Foundation	https://www.python.org/
Mistral Large 2411	Mistral	https://docs.mistral.ai/api
ChatGPT-4o	OpenAI	https://openai.com/index/gpt-4o-system-card
Llama-3.3-70b-versatile	Groq	https://console.groq.com/docs/model/llama-3.3-70b-versatile

**Other**		

Code Repository	–	https://github.com/flaviarodrigues2/article-llms-misinformation

### Method details

#### Data

**Figure 4:**
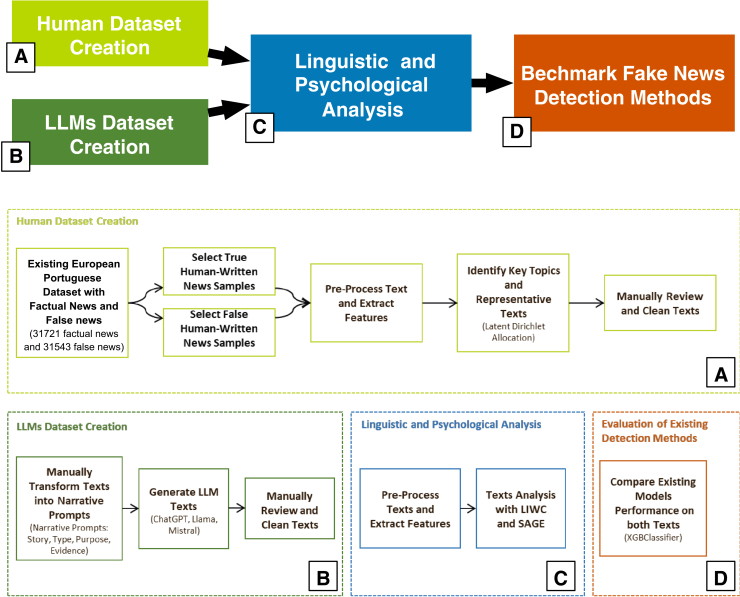
Illustration of the four stages of the methodology pipeline (A) Creation of a human-authored dataset based on European Portuguese news. (B) Generation of a parallel dataset using LLMs based on narrative prompts. (C) Linguistic and psychological analyses using LIWC and SAGE. (D) Evaluation of an existing misinformation detection model. The pipeline setup has been shown. LLMs, large language models; LIWC, linguistic inquiry and word count; SAGE, sparse additive generative model.

Following the steps proposed by^12^ (focused exclusively on the English language and fake news related to COVID-19 and ChatGPT-3.5.) we study news written in the Portuguese language across multiple domains – such as, politics, healthcare, and war – and make use of three state-of-the-art LLMs – GPT-4o, Mistral Large, and Llama 3.3 70B – to examine both factual and false news. In doing so, we investigate the differences across languages, contributing to a more comprehensive understanding of LLMs and misinformation. Figure 4 illustrates the different elements of the pipeline used to generate the datasets and perform the analysis.

#### Human-written dataset

We begin by creating two human-generated datasets: one containing factual news and another containing false news. To that end, we sourced a corpus of European Portuguese factual and false news from.^53^ The dataset, compiled in 2023, comprises 31,721 factual news articles and 31,543 fake news articles from diverse sources, including reputable news websites, websites identified as unreliable, and fact-checking platforms. Nevertheless, it was necessary to perform several pre-processing steps, including correcting data types, handling missing values, removing duplicates, and standardizing source names.

Then, we filtered the dataset to ensure only human-written articles were considered. To that end, and for factual news, we selected articles from traditional media sources – such as SIC Notícias, Diário de Notícias, Expresso, and Jornal de Notícias – authored by professional journalists. For false news, we have excluded short texts (as they often lacked sufficient content or structure), texts from fact-checking sources (as they do not constitute original content), and articles from the “Direita Política” category due to broken or inaccessible source links. At this stage, we were left with 10,000 factual news articles and 6,713 fake news articles.

Since our goal was to work with a smaller sample of articles, and to ensure we sampled articles with an appropriate diversity of topics, we use Latent Dirichlet Allocation (LDA)^59^ to identify latent topic clusters and extract the most representative documents for each topic.^60^^,^^61^ Table 1 provides a topical description of the selected texts, showing the diversity of topics represented. The original Portuguese keywords can be consulted in Table S1. Coherence scores for the factual news dataset and fake news dataset are reported in Figure S1, showing a few notable differences regarding topic stability. In Figure S2, we report inter-topic distances. We then extracted the most representative documents from each topic. In doing so, we produced two refined datasets – of factual and false news – that are further refined by removing titles, links, and incoherent sentences. We then performed a manual review and eliminated news articles that represented near-duplicates, which we qualified as articles covering the same news item and showing only minimal semantic variation, resulting in approximately 200 distinct news articles (half factual, and half false).

A control group of 20 false news articles was manually curated from the same dataset as the human-authored texts to assess the presence of the linguistic patterns under analysis here and potential contamination by LLM-generated content in the human dataset. To further support this assumption, the selection process considered the timeline of generative AI adoption, ensuring that these articles were published before the widespread availability of tools like ChatGPT, released at the end of 2022.^62^ Additionally, the writing style of these texts was examined to confirm consistency with typical human-authored content. These articles were used as a reference point to validate the integrity of the larger fake news sample and, more importantly, to ensure that the large-scale use of AI-generated content did not contaminate the results.

The correlation analysis shown in Figures 2A–4C further validated the consistency between the linguistic characteristics of human-generated fake news and those of the control group, revealing strong positive relationships across all three LLMs. SAGE results reported in Tables S4 and S5 for the control found similar distinguishing patterns between LLM- and human-generated news content. These results indicate that the linguistic patterns found in the broader fake news dataset closely align with those observed in the control group, which consisted of texts verified as human-written before the emergence of generative AI. This strengthens the assumption that the fake news articles in the primary dataset were also authored by humans, reinforcing the validity of the comparison with LLM-generated content, though any residual contamination cannot be excluded with absolute certainty.

#### AI-generated dataset

The second step involves creating an AI-generated counterpart for each human-written news article. The aim is to produce texts concerning the same themes as those written by humans while leveraging the generative creativity of LLMs, and consequently, to examine the linguistic and structural distinctions between AI-generated and human-generated content. To that end, we use three state-of-the-art LLMs with multilingual capabilities: ChatGPT (gpt-4o)^63^^,^^64^; Mistral (mistral-large-2411)^65^^,^^66^^,^^67^; and Llama (llama-3.3-70b-versatile).^68^^,^^69^

Each model was prompted via API using a set of narrative prompts and components derived from the human-written dataset. Building on the Narrative Theory framework^12^^,^^70^ proposes to define discourse through three principal components: type, purpose, and evidence. The type distinguishes whether the text is presented as a news article or a post. The purpose dimension follows journalistic practices, categorizing content as either report, instruction, or commentary, depending on its communicative intent. Evidence refers to the support provided within the text to substantiate its claims. Hence, each article was manually annotated regarding these elements, using only the “Notícia” (news) version under the “type” variable. The remaining components – story, purpose, and evidence – were manually annotated according to the theoretical framework’s definitions. The examples below illustrate the prompts used to generate factual and false news texts.

1. **Prompt Template 1 (Factual News):** Write a news [type] reporting [purpose: report] that non-communicable diseases, such as cardiac problems, cancer, diabetes, and respiratory pathologies, currently surpass infectious diseases and are the main causes of death worldwide [story], according to the World Health Organization (WHO) [evidence].
2. **Prompt Template 2 (False News):** Write a news [type] to give instructions [purpose: instruction] about an old recipe that is being promoted as a natural cure for cancer, including ingredients such as natural honey, aloe vera, and alcohol [story].

The four narrative variables applied in the prompt design are also summarized in Table 2, which provides concise definitions and illustrative examples extracted from these templates.

We opted for a variable-length approach in the text generation process. While other works^71^^,^^72^ use either a fixed-length benchmark dataset or a combination of variable- and fixed-length dataset, for the purposes of our contribution, we chose to focus on variable-length generations, as we considered this closer to the original genre, news articles, which typically represent text of varying length depending on genre, publication type or medium.

AI content generation was conducted under controlled conditions to ensure consistency across all models. Prompts were constructed using a narrative approach, thereby avoiding contamination by sentences, titles and leads from existing, human-written news articles. The generation parameters were standardized to a temperature of 0.7 and a top p of 1.0. The temperature parameter has been associated with the novelty of outputs,^73^ while top-p sampling is proposed as a way to ensure coherence in LLM outputs.^74^ The chosen configuration enables some creativity and diversity in the generated outputs while ensuring alignment with human-written texts.

We conducted several robustness tests regarding the validity of results under the scenario of different text generation parameters. In Table 6, we provide the parameters used for these robustness tests, including the reference value used in the main results. We varied both the temperature and $Top_{p}$ parameters of the models used. We varied the tests by, respectively, holding temperature and $Top_{p}$ constant and varying them both.

Changes in the text output produced by LLMs under different parameter settings did not introduce notable variation in the linguistic fingerprints detected by the methods applied in this article.

Following the generation of the articles, a manual review was conducted to ensure consistency across the dataset: titles and subtitles generated by the LLMs were removed to match the plain text structure of the human-written articles; standard disclaimers and endings – such as “Fim da notícia.” (End of news) or “Nota: Esta notícia é fictícia e foi criada para fins de exemplo.” (Note: This news item is fictitious and was created as an example) – were excluded; location and date headers at the beginning of the texts were removed; formatting markers such as bold and headings were removed, along with double line breaks. These steps ensure that both AI and human-written articles are consistent in format. Table 7 shows English translations of news examples, with the original Portuguese texts available in Table S2.

**Table 7:** Examples of factual and false news texts and the corresponding LLM-generated versions (first two paragraphs)

News Type	Text
Factual news	Non-communicable diseases, such as cardiac conditions, cancer, diabetes, and respiratory pathologies, currently surpass infectious diseases and are the ones that kill the most worldwide, the World Health Organization (WHO) warned this Wednesday. The WHO released this Wednesday a new report and a portal with data from 194 countries about non-communicable diseases and their respective risk factors: smoking, unhealthy diet, harmful use of alcohol, lack of physical activity, and air pollution.
LLM-generated factual news (ChatGPT)	In a revealing report recently published, the World Health Organization (WHO) announced that non-communicable diseases (NCDs), such as cardiac problems, cancer, diabetes, and respiratory pathologies, have surpassed infectious diseases and are now the main causes of death worldwide. This significant change underscores the urgent need for public health strategies focused on the prevention and management of these chronic conditions. According to the data presented, NCDs are responsible for about 71% of all deaths globally. Cardiac problems and cerebrovascular accidents lead the list, followed by cancer, chronic respiratory diseases, and diabetes. Together, these conditions represent a monumental challenge for health systems, especially in low- and middle-income countries, where nearly 85% of premature deaths (between 30 and 69 years old) caused by NCDs occur.
False news	According to the latest study carried out in 2015 by Ernst & Young on fraud and corruption in various countries around the world, there are still high levels of corruption spread across the different continents. In the European case, the news is not good for Portugal, which appears in this ranking as one of the most corrupt countries in Europe, occupying fourth place. The truth is that when Portuguese workers were surveyed, the study reveals that at least 83% consider that corruption or bribery practices in Portugal happen in a widespread manner, being almost a common practice.
LLM-generated false news (Llama)	According to a recent study published by an international anti-corruption organization, Portugal was classified as the fourth most corrupt country in Europe. This alarming classification places Portugal behind only three other European countries in terms of perceived corruption levels. The study, which analyzed data from 27 European countries, used a combination of indicators, including perception of corruption, effectiveness of institutions, and protection of human rights. The results show that Portugal presents significant levels of corruption in sectors such as public administration, justice, and the economy.

#### Linguistic characteristics analysis

To perform a linguistic comparison between AI-generated content and human-generated content, we first conducted descriptive analyses to explore fundamental text-level differences. We then employ two primary approaches: the Linguistic Inquiry and Word Count (LIWC) and the Sparse Additive Generative Model (SAGE).

LIWC^75^ is a text analysis tool that quantifies words in approximately 80 meaningful categories, representing linguistic, psychological, and social processes. This allows for nuanced analysis in areas such as emotional expression, cognitive styles, and interpersonal communication. The method processes each text word by word, calculating the percentage of words in each LIWC category.^75^^,^^76^ Here, we use the Portuguese-adapted LIWC dictionary, BP-LIWC2015,^77^ that contains 73 linguistic and psychological categories and 14,459 words. This version was developed explicitly for Brazilian Portuguese and has been empirically evaluated in text classification tasks across multiple datasets and algorithms.^78^^,^^79^ A lexical adaptation was performed to align key terms to European Portuguese. In total, we consider five LIWC categories, which include a total of 25 sub-categories, organized as follows:

**Informal and Netspeak Attributes** – Capture casual and online-specific language, including informal expressions (e.g., “uhm”, “aiai”) and netspeak (e.g., “tweet”, emojis), often found in social media or messaging platforms.

**Emotional and Affective Attributes** – Encompass words related to emotional expression, including positive feelings (e.g., “amigável”, “amar”) and negative states like anxiety, anger, or sadness.

**Cognitive Attributes** – Reflect mental activities such as reasoning and reflection. This includes indicators of insight (e.g. “pensar”), causation (e.g. “porque”), discrepancy (e.g. “deveria”), tentativeness (e.g. “talvez”), certainty (e.g. “sempre”), and differentiation (e.g. “contudo”).

**Perceptual Attributes** – Capture references to sensory experience, highlighting the speaker’s focus on seeing (e.g., “olhar”), hearing (e.g., “ouvir”), or feeling (e.g., “peso”).

**Motivational and Drive Attributes** – Identify underlying motivations and goals through linguistic markers of affiliation (e.g., “aliança”), achievement (e.g., “sucesso”), power (e.g., “controlar”), reward (e.g., “ganhar”), and risk (e.g., “esconder”).

Linguistic category analysis was based on the proportion of words in each LIWC category per text, normalized by the total word count, and the relative difference (D%) was calculated by comparing AI-generated texts to the human baseline for each category.

Moreover, we use SAGE^80^ to identify terms—words or n-grams—that are particularly characteristic of one group of texts compared to another. SAGE estimates deviations in the log-frequency of words relative to a background lexical distribution. It applies a self-tuned regularization parameter to emphasize salient lexical differences while reducing noise from overly common or rare terms. Here, we use SAGE to compare, separately, human-authored factual and false news datasets with their AI-generated counterparts. Before performing the SAGE analysis, the texts are preprocessed into unigrams, bigrams, and trigrams, with stop words and isolated numbers removed. For each pairwise comparison between false and factual news, the model generates salience scores (η) for each term in the shared vocabulary, highlighting which lexical items are most strongly associated with either human- or AI-generated content.

The salience scores (η) correspond to the log-ratio of their frequency in the human-generated corpus distribution versus the distribution in the AI-generated corpus. Hence, in such pairwise comparisons, the salience score captures how much more/less frequent each term is when comparing the two corpora. As such, in our case, the exp(η) indicates the relative frequency of a term in the human-generated corpus in comparison to the AI-generated one. Positive salience scores (η>0) indicate that terms are more frequent in human-generated content and, conversely, negative scores (η<0) indicate that terms are more frequent in AI-generated texts.

The terms with the most extreme salience scores reveal the ones that are, comparatively, more distinctive/unique in each of the sources.

#### Fake news detection model

This section assesses the efficacy of an existing detection model in identifying AI-generated misinformation. For this purpose, we implement the best-performing model proposed by^53^ in European Portuguese. The authors developed several machine learning and deep learning models for misinformation detection to integrate them into end-user tools, such as browser extensions and smartphone applications. Among the models evaluated, the XGBoost classifier achieved the highest performance for European Portuguese with hyperparameters *colsample bytree* of 0.8, *learning rate* of 0.2, *max depth* of 5, *number of estimators* of 300, and *subsample* of 1, achieving an F1-score of 0.957.

In the present study, we train the same model, using the XGBoost library in Python, with the same hyperparameters on the original dataset. Only previously unseen examples from this dataset—103 fake and 103 real human-written texts—are used for testing. Additionally, a new set of 103 fake and 103 real texts generated by large language models is introduced to assess the model’s ability to detect AI-generated content.

All texts were processed using the same pre-processing steps, including tokenization, stopword removal, lemmatization, part-of-speech tagging to extract counts of nouns, verbs, adjectives, and adverbs, and sentiment scoring with the VADER sentiment analyzer.^81^ The Source feature is excluded to avoid bias, since the LLM-generated texts do not contain this metadata. These features, together with the pre-processed text, are concatenated and transformed into TF-IDF vectors to serve as inputs for the XGBoost classifier.

The model’s performance is evaluated through accuracy, precision, recall, and F1-score metrics. Furthermore, we performed a chi-square $\left(\chi^{2}\right)$ test to determine whether the differences in detection between human and AI-generated misinformation are statistically significant. This test assesses whether there is a significant association between the source of the misinformation (human vs AI) and the model’s classification outcomes, based on the distribution of correct and incorrect predictions.

### Quantification and statistical analysis

In our LIWC analysis, we employ the Wilcoxon signed-rank test to test differences between the human baseline of the five LIWC categories and LLM-generated text, with Benjamini-Hochberg correction applied to adjust for multiple comparisons. Throughout the tables, statistical significance is denoted as follows: p<0.05(^∗^); p<0.01(^∗∗^); p<0.001(^∗∗∗^). The absence of an asterisk indicates that the coefficient is not significant at any of these levels.
