# Supplementary material for: A linguistic comparison between human- and AI-generated content
Source: iScience. 2026 Feb 12;29(3):114976. doi: 10.1016/j.isci.2026.114976 (PMC12969083; doi:10.1016/j.isci.2026.114976)
Supplement: Document S1. Figures S1–S6 and Tables S1–S13 [file mmc1.pdf]

iScience, Volume *29*

Supplemental information

A linguistic comparison

between human- and AI-generated content

Flávia A. Rodrigues, Niclas F. Sturm, and Flávio L. Pinheiro

1 Portuguese Topical Description

Table S1: Original version of Table 4 in Portuguese language.

Topic	**True News**		**Fake News**	
	Top Keywords	Frequency	Top Keywords	Frequency
1	governo, medida, euro	7.02%	ano, euro, banco	9.52%
2	caso, tribunal, processo	4.42%	mulher, vida, casa	13.29%
3	governo, poder, partido	11.54%	Portugal, país, poder	7.51%
4	Lisboa, cidade, local	6.44%	água, poder, praia, casa	5.13%
5	ano, vítima, autoridade	6.11%	km_radar, Lisboa, estrada	3.16%
6	nato, turquia, incêndio	2.14%	poder, pessoa, bom	9.21%
7	ucrânia, russo, rússia	6.93%	carro, poder, veículo	4.39%
8	poder, saúde, ano	13.98%	empresa, Portugal, estado	7.16%
9	poder, ano, pessoa	8.43%	tribunal, arguido, massa	1.90%
10	país, europeu, ue	6.98%	sistema, modelo, tecnologia	2.22%
11	poder, Estados Unidos, mundo	4.84%	governo, deputado, euro	5.18%
12	empresa, euro, ano	9.57%	médico, hospital, dia, poder	4.88%
13	português, Portugal, poder	7.04%	mundo, pessoa, vídeo, ano	8.71%
14	jogo, equipa, ano	4.54%	português, jogador, nome	3.58%
15	-	-	criança, pai, ano	8.83%
16	-	-	poder, corpo, dia	5.35%

2 Example Texts

Table S2: Original Portuguese Version of the First Two Paragraphs of the Example Texts, extending Table 6.

News Type	Text
Factual News	Doenças não transmissíveis, como as do foro cardíaco, cancro, diabetes e patologias respiratórias, superam atualmente as enfermidades infecciosas e são as que mais matam no mundo, alertou esta quarta-feira a Organização Mundial da Saúde (OMS).<br>A OMS divulgou esta quarta-feira um novo relatório e um portal com dados de 194 países sobre doenças não transmissíveis e respetivos fatores de risco: tabagismo, alimentação não saudável, uso nocivo de álcool, falta de atividade física e poluição do ar.
LLM-Generated Factual News (ChatGPT)	Em um relatório revelador divulgado recentemente, a Organização Mundial da Saúde (OMS) anunciou que as doenças não transmissíveis (DNTs), como problemas cardíacos, cancro, diabetes e patologias respiratórias, ultrapassaram as doenças infecciosas e agora são as principais causas de morte em todo o mundo. Esta mudança significativa sublinha a necessidade urgente de estratégias de saúde pública focadas na prevenção e gestão dessas condições crônicas.<br>De acordo com os dados apresentados, as DNTs são responsáveis por cerca de 71% de todas as mortes a nível global. Problemas cardíacos e acidentes vasculares cerebrais lideram a lista, seguidos pelo cancro, doenças respiratórias crônicas e diabetes. Juntas, essas condições representam um desafio monumental para os sistemas de saúde, especialmente em países de baixa e média renda, onde quase 85% das mortes prematuras (entre 30 e 69 anos) causadas por DNTs ocorrem.
False News	Segundo o último estudo realizado em 2015 pela Ernst & Young, sobre fraude e corrupção em vários países do mundo, ainda existem fortes índices de corrupção espalhados pelos vários continentes. No caso Europeu, as notícias não são boas para Portugal, que aparece neste ranking como sendo um dos países mais corruptos da Europa, ocupando o quarto lugar.<br>A verdade é que ao inquirirem trabalhadores portugueses, o estudo revela que, pelo menos 83% considera que as práticas de corrupção ou de suborno em Portugal acontecem de forma generalizada, sendo quase uma prática comum.
AI-Generated False News (Llama)	De acordo com um estudo recente publicado por uma organização internacional de combate à corrupção, Portugal foi classificado como o quarto país mais corrupto da Europa. Essa classificação alarmante coloca Portugal atrás de apenas três outros países europeus em termos de níveis de corrupção percebida.<br>O estudo, que analisou dados de 27 países europeus, utilizou uma combinação de indicadores, incluindo a percepção da corrupção, a eficácia das instituições e a proteção dos direitos humanos. Os resultados mostram que Portugal apresenta níveis significativos de corrupção em setores como a administração pública, a justiça e a economia.

3 Additional SAGE results

Table S3: Original Portuguese keywords distinguishing human-authored and AI-generated texts identified by SAGE, by model and veracity category. Keywords occurring in at least two models for LLM keywords and keywords occurring at least twice for human keywords are in bold.

**Modelo**	**Tipo de Notícia**	**Palavras-chave LLM**	**Palavras-chave Humanas**
**ChatGPT**	Factual News	**mas também** (-3.89); à medida (-3.64); enquanto isso (-3.33); na região (-3.24); **além disso** (-3.24); não apenas (-3.14); incidente (-3.24); possam (-3.14); reforçar (-3.14); **em relação** (-3.03)	**vai** (3.89); face (3.46); **conta** (3.40); **novembro** (3.40); **vão** (3.33); **referiu** (3.26); julho (3.26); presidente da república (3.19); **florida** (3.19); **euros m2** (3.19)
**ChatGPT**	False News	**importância** (-4.31); **para garantir** (-3.79); **mas também** (-3.48); **políticas** (-3.05); gestão (-3.05); não apenas (-2.96); do país (-2.96); ao longo (-2.96); este signo (-2.86); **incluindo** (-2.75)	**por isso** (4.14); logo (3.51); **os seus** (3.43); **são pessoas** (3.35); **km** (3.35); **vai** (3.32); **porque** (3.28); salário (3.26); pessoa (3.26); sangue (3.16)
**Mistral**	Factual News	é crucial (-3.75); ministério da (-3.47); transparência (-3.39); mercado imobiliário (-3.30); **mas também** (-3.21); adesão da suécia (-3.21); um momento (-3.21); monitorar (-3.21); preocupações (-3.21); comunidade internacional (-3.11)	comunicado (3.60); **novembro** (3.42); **conta** (3.42); **porque** (3.36); **referiu** (3.29); **florida** (3.21); **euros m2** (3.21); terceiro (3.21); secretário-geral (3.13); suécia nato (3.04)
**Mistral**	False News	**para garantir** (-4.42); **importância** (-4.28); **incluindo** (-3.42); para evitar (-3.22); comentou (-3.06); **mas também** (-2.98); ainda não (-2.98); tem gerado (-2.89); se você (-2.89); **políticas** (-2.79)	**por isso** (4.18); **vai** (4.05); as pessoas (3.55); **vão** (3.55); depósito (3.55); **os seus** (3.47); **são pessoas** (3.39); **porque** (3.32); mês (3.30); pois (3.25)
**Llama**	Factual News	**além disso** (-4.28); marcelo rebelo (-3.70); **em relação** (-3.55); um sinal (-3.55); pacientes (-3.55); vacinação contra a (-3.55); nos próximos dias (-3.47); é um passo (-3.47); uma experiência (-3.47); **mas também** (-3.39)	marcelo rebelo de (3.65); comunicado (3.55); quinta-feira (3.49); grupo (3.43); donald trump (3.37); **novembro** (3.37); presidente da república (3.16); **euros m2** (3.16); maio (3.07); quarta-feira (3.07)
**Llama**	False News	um lembrete (-4.09); **importância** (-3.84); é fundamental (-3.59); **incluindo** (-3.59); os consumidores (-3.53); promover (-3.47); o acidente (-3.33); pode ter (-3.26); do partido (-3.10); **mas também** (-3.01)	**por isso** (4.12); **os seus** (3.42); **são pessoas** (3.34); **km** (3.34); quase (3.34); neste (3.34); mil (3.15); avisar (3.15); acontecer (3.15); radar (3.15)

Table S4: English Version of SAGE Keywords for the Control Group, related to analysis in Figure 4.

Model	LLM Keywords	Human Keywords
**ChatGPT**	but also (-3.05); importance (-2.32); not only (-1.91); simple (-1.91); reduce (-1.91); not just (-1.62); those who (-1.19); long (-1.19); promote (-1.19); changes (-1.19)	stay (2.81); all the (2.67); period (2.50); many (2.50); from sporting (2.50); in this (2.50); subject (2.30); everything (2.30); in lisbon (2.30); other (2.30)
**Mistral**	dr (-2.45); avoid (-2.10); but also (-2.10); ana (-2.10); crisis (-1.88); alcoholete (-1.58); public health (-1.58); investigate (-1.58); of the study (-1.10); electric vehicles (-1.10)	state (3.07); year (3.07); patients (2.96); home (2.83); hospital (2.83); days (2.69); all (2.52); from sporting (2.52); age (2.52); crimes (2.52)
**Llama**	reduce (-3.54); conclusion (-3.13); it's important (-3.03); incident (-2.80); avoid (-2.80); ensure (-2.66); discovery (-2.51); of the sea (-2.33); management (-2.33); sporting cp (-2.12)	state (2.99); home (2.75); stay (2.75); because (2.61); cases (2.61); from sporting (2.44); all (2.44); many (2.44); a group (2.44); millions (2.38)

Table S5: Original Portuguese Version of SAGE Keywords for the Control Group, related to analysis in Figure 4.

Modelo	Palavras-chave LLM	Palavras-chave Humanas
**ChatGPT**	mas também (-3.05); importância (-2.32); não apenas (-1.91); simples (-1.91); reduzir (-1.91); não só (-1.62); aqueles que (-1.19); longa (-1.19); promover (-1.19); mudanças (-1.19)	ficar (2.81); todos os (2.67); período (2.50); muitas (2.50); do sporting (2.50); nesta (2.50); assunto (2.30); tudo (2.30); em lisboa (2.30); outro (2.30)
**Mistral**	dr (-2.45); evitar (-2.10); mas também (-2.10); ana (-2.10); crise (-1.88); alcoohete (-1.58); saúde pública (-1.58); investigar (-1.58); do estudo (-1.10); veículos elétricos (-1.10)	estado (3.07); ano (3.07); doentes (2.96); casa (2.83); hospital (2.83); dias (2.69); toda (2.52); do sporting (2.52); idade (2.52); crimes (2.52)
**Llama**	reduzir (-3.54); conclusão (-3.13); é importante (-3.03); incidente (-2.80); evitar (-2.80); garantir (-2.66); descoberta (-2.51); do mar (-2.33); gestão (-2.33); sporting cp (-2.12)	estado (2.99); casa (2.75); ficar (2.75); porque (2.61); casos (2.61); do sporting (2.44); tudo (2.44); muitas (2.44); um grupo (2.44); milhões (2.38)

4 LDA – Additional Results

4.1 Coherence Scores

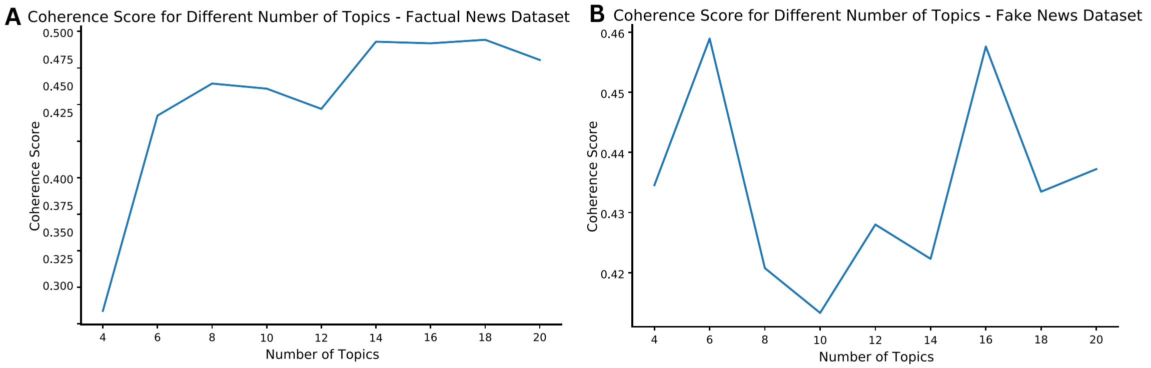

Figure S1: Coherence Score for LDA topic model reported in Table 4 of the main article. **Panel A**: Coherence scores for the factual news dataset. **Panel B**: Coherence scores for the fake news dataset. A few notable differences emerge between the model applied to the factual news dataset and that applied to the fake news dataset. Although the coherence score reaches a plateau in the case of factual news, there are certain gyrations in the coherence score of the LDA model on the fake news dataset. This might suggest that factual news has a higher degree of topic stability and that the plateau occurs when the major themes have been covered, leaving only such topics that can be considered ancillary topics of existing ones. The coherence scores on the fake news dataset move in a much narrower band (between 0.41 and 0.46) and lack a clear trend regarding the number of topics, suggesting that the model is only able to identify broad narratives that cannot consistently be decomposed into sub-topics.

4.2 Inter-topic distances

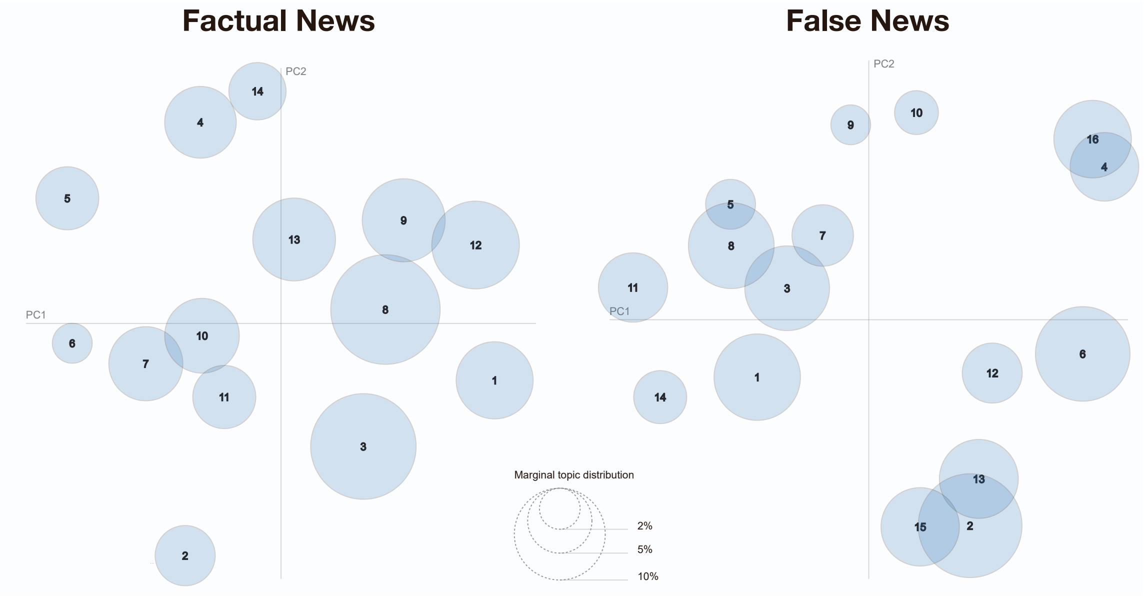

Figure S2: Inter-topic distance for LDA topic model reported in Table 4 of the main article.

5 LLM Text generation – Robustness

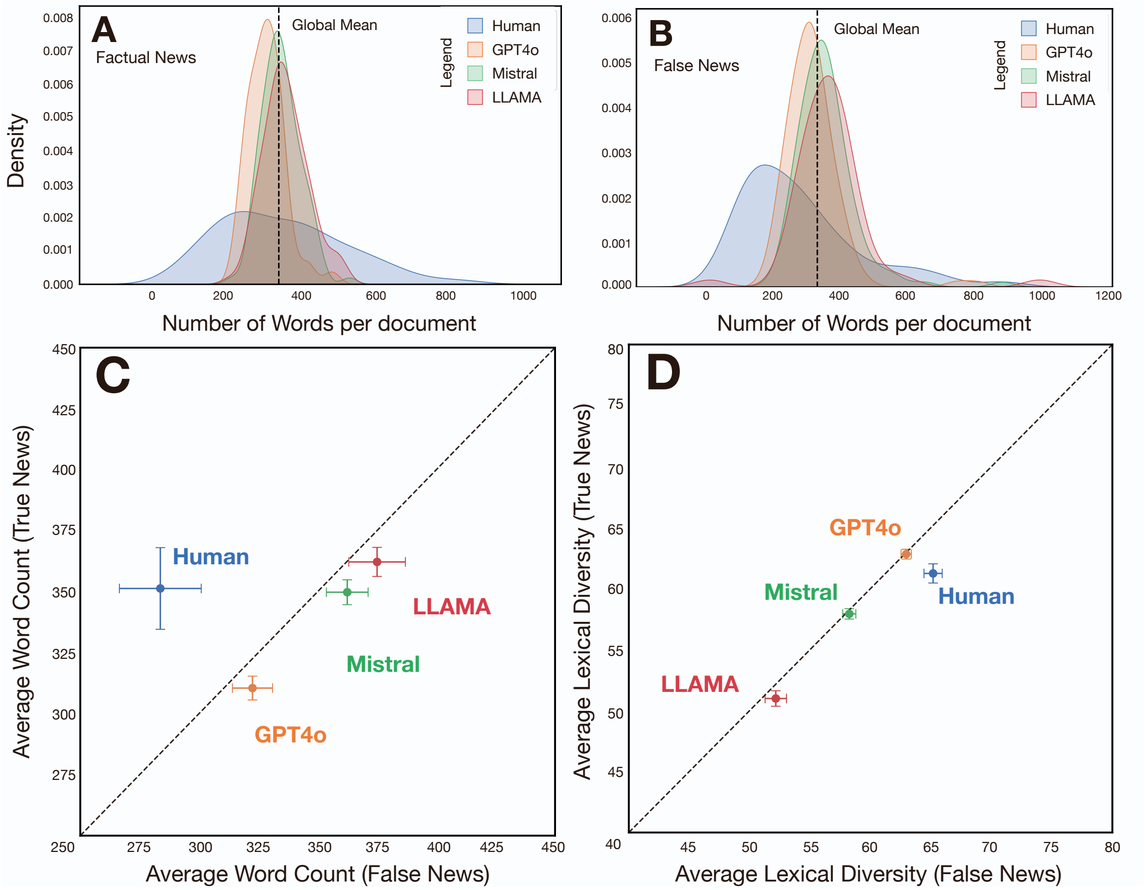

Figure S3: **Temperature: 0.5; Top$_p$: 1**, reproduction of Figure 1 with changed generation parameters. Descriptive comparison between the documents produced by Humans and LLMs. Panels A and B compare the distribution in terms of word count (length) of the articles with factual (A) and false (B) news. Panels C and D compare both factual and false news by source – Humans or LLMs – in terms of average document size (A) but also lexical diversity (D). Word Count (Factual/Fake News) and Lexical Diversity (Factual/Fake News) are represented as mean word count/lexical diversity ± standard deviation.

Table S6: **Temperature: 0.5; Top$_p$: 1**, reproduction of Table 2 with changed generation parameters. Salient Keywords Distinguishing Human-Authored and AI-Generated Texts Identified by SAGE. Expressions appearing twice between models are highlighted in bold.

**Model**	**News Type**	**LLM Keywords**	**Human Keywords**
**ChatGPT**	Factual News	ainda não (-3.10); **à medida** (-3.29); **especialmente** (-3.38); **equipe** (-2.99); **mas também** (-3.86); oferece (-3.10); possam (-3.38); reforçar (-3.10); sua posição (-3.29)	**vai** (3.93); face (3.49); tendo (3.49); aumentou (3.37); julho (3.30); **referiu** (3.30); **presidente da república** (3.22); **euros m2** (3.22); **perante** (3.14)
**ChatGPT**	False News	**importância** (-4.20); para garantir (-3.82); **mas também** (-3.40); enquanto isso (-3.33); gestão (-3.26); garantindo (-3.19); para evitar (-3.19); ao longo (-2.94); não só (-2.94); do país (-2.84)	porque (4.39); **por isso** (4.15); **vai** (4.03); nada (3.66); fazer (3.64); **as pessoas** (3.52); idade (3.45); **os seus** (3.45); **km** (3.36); **são pessoas** (3.36)
**Mistral**	Factual News	ainda mais (-3.51); para mais informações (-3.35); uma série (-3.26); os desafios (-3.17); é crucial (-3.17); **especialmente** (-3.17); a comunidade internacional (-3.17); marcelo rebelo (-3.17); **equipe** (-3.07); possam (-3.07)	marcelo rebelo de (3.74); novembro (3.46); conta (3.46); **vão** (3.39); **referiu** (3.32); **euros m2** (3.25); florida (3.25); **presidente da república** (3.25); secretário-geral (3.16); **perante** (3.16)
**Mistral**	False News	**importância** (-4.15); **mas também** (-3.47); críticas (-2.98); é crucial (-2.89); perto (-2.89); se tu (-2.79); pesquisa (-2.79); justiça (-2.79); curiosidade (-2.79); polémica (-2.79)	**por isso** (4.18); bastante (3.69); **vão** (3.55); **as pessoas** (3.55); depósito (3.55); **os seus** (3.47); **são pessoas** (3.39); **km** (3.39); acontecer (3.20); av (3.20)
**Llama**	Factual News	além disso (-4.18); um dos principais (-3.83); **equipe** (-3.77); marcelo rebelo (-3.70); um lembrete (-3.57); presidente marcelo rebelo (-3.49); economia portuguesa (-3.41); **mas também** (-3.41); também destacou (-3.41); **à medida** (-3.41)	marcelo rebelo de (3.69); quinta-feira (3.53); tendo (3.47); face (3.47); novembro (3.41); **vão** (3.34); **referiu** (3.27); cerca (3.25); **euros m2** (3.20); **presidente da república** (3.20)
**Llama**	False News	um lembrete (-4.02); **importância** (-3.98); é fundamental (-3.66); incluindo (-3.55); **mas também** (-3.36); do país (-3.36); é importante (-3.29); gestão (-3.22); um medo profundo (-3.22); promover (-3.14)	por isso (4.15); bastante (3.66); logo (3.52); **os seus** (3.44); **são pessoas** (3.36); **km** (3.36); quase (3.36); profissional (3.27); acontecer (3.17); manhã (3.17)

Table S7: **Temperature: 0.5; Top$_p$: 1**, reproduction of Table 1 with changed generation parameters. Linguistic differences between human- and AI-generated texts across all five LIWC categories. Each cell shows the relative difference (Δ%) between each LLM (GPT-4o, Mistral, Llama) and the human baseline, along with the significance levels obtained using the Wilcoxon signed-rank test. Positive values indicate higher usage by LLMs; negative values indicate lower usage. Significance levels after Benjamini-Hochberg correction for multiple comparisons: $p < 0.05(^*)$, $p < 0.01(^{**})$, $p < 0.001$ $(^{***})$.

**LIWC Category**	**Factual News**			**Fake News**		
	**ChatGPT**	**Mistral**	**Llama**	**ChatGPT**	**Mistral**	**Llama**
**Informal and Netspeak Attributes**						
informal (Informal Language)	**-72.55%*****	**-61.62%*****	**-74.92%*****	**-46.48%*****	**-51.14%*****	**-55.63%*****
netspeak (Netspeak)	-53.44%	**-81.36%***	**-100.00%***	**-79.23%****	**-62.67%***	**-79.22%***
**Emotional and Affective Attributes**						
affect (Affect)	**79.41%*****	**88.92%*****	**114.48%*****	**54.70%*****	**55.91%*****	**70.94%*****
posemo (Positive Emotions)	**90.44%*****	**105.26%*****	**135.23%*****	**84.90%*****	**83.56%*****	**85.88%*****
negemo (Negative Emotions)	21.37%	20.92%	39.75%	14.10%	0.46%	24.26%
anx (Anx)	3.40%	14.56%	9.67%	**-52.01%*****	**-57.78%*****	**-51.98%*****
anger (Anger)	-17.51%	-14.63%	-12.31%	**-23.81%****	**-35.07%****	**-35.71%****
sad (Sad)	**-40.21%****	**-42.30%****	**-61.03%*****	-16.81%	**-27.23%****	7.15%
**Cognitive Attributes**						
cogproc (Cognitive Processes)	**51.10%*****	**54.55%*****	**60.41%*****	22.47%	28.03%	**50.03%****
insight (Insight)	47.16%	29.33%	49.68%	**59.40%***	44.72%	56.48%
cause (Causal)	**67.01%*****	**65.51%*****	**84.17%*****	**37.37%***	**41.57%***	**46.14%****
discrep (Discrepancies)	15.70%	16.09%	15.51%	**-8.88%****	**-10.89%*****	17.50%
tentat (Tentative)	47.87%	52.66%	68.49%	**-8.47%****	7.61%	41.40%
certain (Certainty)	**137.61%*****	**160.13%*****	**100.58%*****	34.93%	**44.32%****	9.83%
differ (Differentiation)	11.45%	19.38%	24.37%	**2.95%****	8.04%	49.41%
**Perceptual Attributes**						
percept (Perceptual Processes)	35.54%	59.23%	47.94%	-1.44%	12.67%	7.32%
see (See)	59.04%	50.11%	34.00%	40.02%	67.46%	23.71%
hear (Hear)	**-51.16%*****	**-38.60%****	-12.59%	**-70.43%*****	**-49.61%*****	**-24.13%****
feel (Feel)	-8.73%	**-19.97%****	1.54%	**-34.17%*****	**-32.74%*****	**-19.56%****
**Motivational and Drive Attributes**						
drives (Drives)	**43.39%*****	**48.67%*****	**55.74%*****	**37.93%*****	**39.04%*****	**49.63%*****
affiliation (Affiliation)	**70.37%*****	34.64%	**42.01%***	24.57%	11.48%	21.43%
achieve (Achievement)	**121.89%*****	**127.08%*****	**132.66%*****	**65.90%*****	**58.39%*****	**74.33%*****
power (Power)	20.51%	25.47%	**31.58%***	**70.18%*****	**54.30%****	**72.96%*****
reward (Reward)	**66.01%*****	**96.48%*****	**94.75%*****	**38.31%***	**54.60%*****	**53.18%****
risk (Risk)	**82.42%*****	**95.10%*****	**116.42%*****	54.47%	**61.38%*****	**78.82%*****

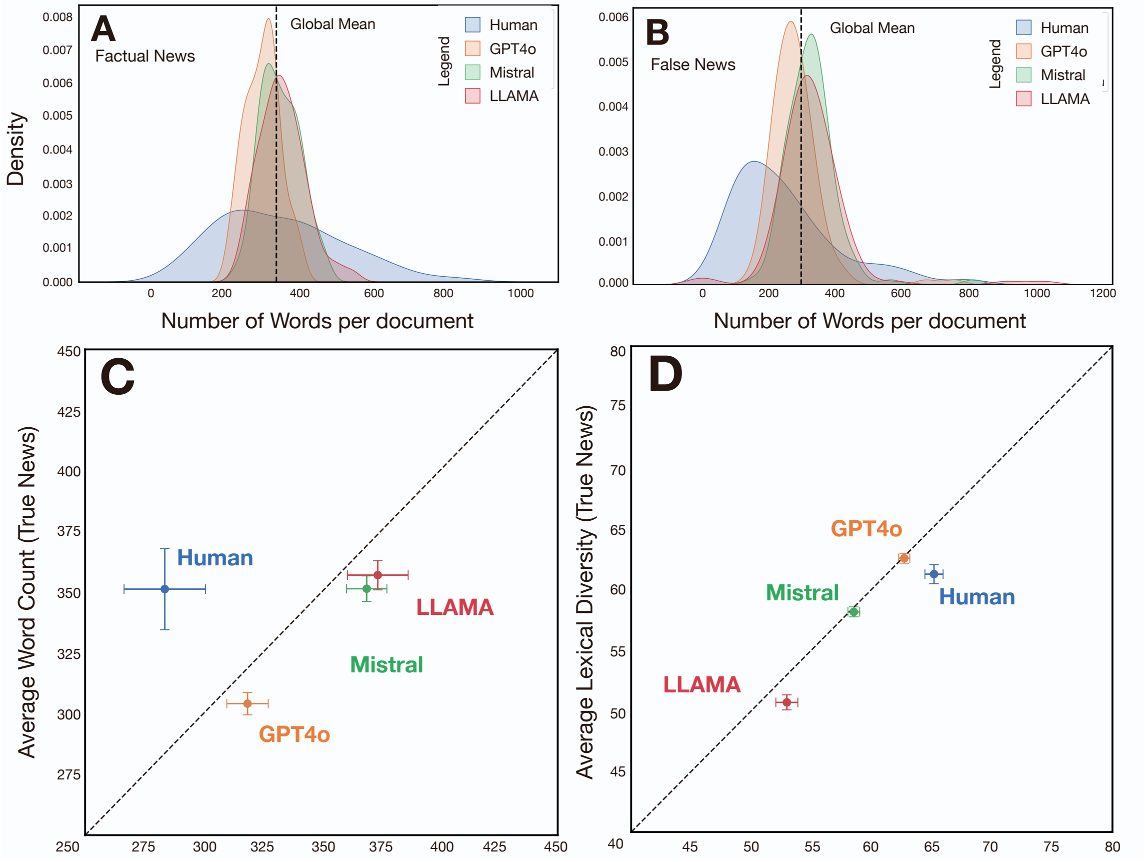

Figure S4: **Temperature: 0.7; Top$_p$: 0.5**, reproduction of Figure 1 with changed generation parameters. Descriptive comparison between the documents produced by Humans and LLMs. Panels A and B compare the distribution in terms of word count (length) of the articles with factual (A) and false (B) news. Panels C and D compare both factual and false news by source – Humans or LLMs – in terms of average document size (A) but also lexical diversity (D). Word Count (Factual/Fake News) and Lexical Diversity (Factual/Fake News) are represented as mean word count/lexical diversity ± standard deviation.

Table S8: **Temperature: 0.7; Top$_p$: 0.5**, reproduction of Table 2 with changed generation parameters. Salient Keywords Distinguishing Human-Authored and AI-Generated Texts Identified by SAGE. Expressions appearing twice between models are highlighted in bold.

**Model**	**News Type**	**LLM Keywords**	**Human Keywords**
**ChatGPT**	Factual News	**mas também** (-3.84); entre os (-3.84); possam (-3.42); enquanto isso (-3.34); gastronomia (-3.15); **além disso** (-3.15); estratégias (-3.15); na região (-3.04); incidente (-3.04); à medida (-2.92)	conta (3.39); **novembro** (3.39); **quase** (3.39); **vão** (3.33); **referiu** (3.26); disse (3.25); vai (3.19); **euros m2** (3.18); **presidente da república** (3.18); secretário-geral (3.10)
**ChatGPT**	False News	**importância** (-4.12); **mas também** (-3.79); para garantir (-3.74); especialistas em (-3.46); à medida (-3.18); **promover** (-3.09); enquanto isso (-3.09); políticas (-3.00); garantindo (-3.00); gestão (-2.80)	**por isso** (4.12); diz (3.74); **porque** (3.66); depósito (3.49); **as pessoas** (3.49); **vão** (3.49); **logo** (3.49); **os seus** (3.41); km (3.33); **quase** (3.33)
**Mistral**	Factual News	marcelo rebelo (-4.22); especialmente (-3.74); ministério da (-3.48); transparência (-3.32); para mais informações (-3.32); pacientes (-3.24); é crucial (-3.24); comunidade internacional (-3.14); **além disso** (-3.04); uma série (-3.04)	**marcelo rebelo de** (3.76); tendo (3.54); face (3.54); **porque** (3.41); **referiu** (3.34); dia (3.33); vai (3.28); **euros m2** (3.26); **presidente da república** (3.26); maio (3.18)
**Mistral**	False News	**importância** (-4.20); **incluindo** (-3.30); do país (-3.24); críticas (-3.09); entre os (-3.01); comentou (-2.84); justiça (-2.84); comunidade local (-2.74); políticas (-2.74); ainda não (-2.63)	**por isso** (4.21); **porque** (3.76); bastante (3.72); **logo** (3.58); **os seus** (3.50); **são pessoas** (3.42); dizer (3.42); novo (3.33); av (3.23); radar (3.23)
**Llama**	Factual News	**além disso** (-4.11); **promover** (-3.65); um dos principais (-3.65); **mas também** (-3.58); equipe (-3.51); em todo (-3.43); privatização (-3.43); não apenas (-3.25); é vista (-3.25); presidente marcelo rebelo (-3.15)	**marcelo rebelo de** (3.68); comunicado (3.57); quinta-feira (3.52); grupo (3.46); **novembro** (3.40); **vão** (3.33); **referiu** (3.26); fevereiro (3.26); julho (3.26); cada (3.18)
**Llama**	False News	**importância** (-3.90); **incluindo** (-3.90); um medo profundo (-3.61); é fundamental (-3.55); é importante (-3.43); **promover** (-3.37); são conhecidos por (-3.37); é uma planta (-3.37); é um lembrete (-3.30); **mas também** (-3.30)	**por isso** (4.14); faz (3.71); todos os (3.71); **porque** (3.69); bastante (3.65); depósito (3.51); **as pessoas** (3.51); **os seus** (3.44); **são pessoas** (3.36); **quase** (3.36)

Table S9: **Temperature: 0.7; Top$_p$: 0.5**, reproduction of Table 1 with changed generation parameters. Linguistic differences between human- and AI-generated texts across all five LIWC categories. Each cell shows the relative difference (Δ%) between each LLM (GPT-4o, Mistral, Llama) and the human baseline, along with the significance levels obtained using the Wilcoxon signed-rank test. Positive values indicate higher usage by LLMs; negative values indicate lower usage. Significance levels after Benjamini-Hochberg correction for multiple comparisons: $p < 0.05(^*)$, $p < 0.01(^{**})$, $p < 0.001$ (***).

**LIWC Category**	**Factual News**			**Fake News**		
	**ChatGPT**	**Mistral**	**Llama**	**ChatGPT**	**Mistral**	**Llama**
**Informal and Netspeak Attributes**						
informal (Informal Language)	**-66.53%*****	**-54.87%****	**-70.89%*****	**-51.86%*****	**-50.43%*****	**-61.65%*****
netspeak (Netspeak)	-37.41%	-34.79%	**-81.97%***	**-83.35%*****	**-67.99%****	**-80.01%***
**Emotional and Affective Attributes**						
affect (Affect)	**93.62%*****	**92.21%*****	**115.41%*****	**46.98%****	**63.56%*****	**51.52%*****
posemo (Positive Emotions)	**102.34%*****	**96.63%*****	**129.35%*****	**66.16%*****	**72.74%*****	**69.48%*****
negemo (Negative Emotions)	29.89%	31.37%	49.87%	12.12%	15.49%	23.59%
anx (Anx)	8.97%	-26.95%	-2.90%	**-42.59%***	**-36.25%***	**-47.92%****
anger (Anger)	-13.54%	-16.92%	**-24.29%***	**-34.21%***	**-41.37%****	**-14.79%***
sad (Sad)	-14.69%	-17.26%	**-40.03%****	7.17%	**-27.23%****	17.80%
**Cognitive Attributes**						
cogproc (Cognitive Processes)	**43.86%*****	**50.86%*****	**61.00%*****	21.64%	22.30%	43.09%
insight (Insight)	27.12%	34.67%	**62.93%***	40.40%	41.34%	49.34%
cause (Causal)	**55.86%*****	**55.45%*****	**67.80%*****	**36.53%***	**35.24%***	**44.51%****
discrep (Discrepancies)	22.18%	15.03%	**39.13%****	**-9.54%****	**-5.09%****	**-3.35%****
tentat (Tentative)	46.20%	48.98%	**86.51%***	**-7.98%****	6.39%	9.93%
certain (Certainty)	**127.80%*****	**163.16%*****	**102.39%*****	27.20%	36.90%	20.00%
differ (Differentiation)	32.94%	21.68%	33.08%	7.94%	**2.48%****	32.46%
**Perceptual Attributes**						
percept (Perceptual Processes)	16.75%	34.10%	38.27%	1.46%	4.35%	0.97%
see (See)	56.11%	49.65%	36.86%	33.52%	21.57%	**6.37%***
hear (Hear)	**-53.20%*****	**-39.39%*****	-7.21%	**-65.83%*****	**-46.06%*****	**-23.98%****
feel (Feel)	**-23.13%****	**-22.65%*****	**-11.50%***	**-29.99%*****	**-30.41%****	**-29.46%*****
**Motivational and Drive Attributes**						
drives (Drives)	**43.68%*****	**41.89%*****	**53.60%*****	**36.58%*****	**37.20%*****	**48.81%*****
affiliation (Affiliation)	**66.32%*****	**52.99%***	38.93%	7.05%	15.98%	22.72%
achieve (Achievement)	**95.42%*****	**85.93%*****	**128.72%*****	**65.81%*****	**58.39%*****	**74.33%*****
power (Power)	19.82%	25.47%	**31.58%***	**70.18%*****	**54.30%****	**72.96%*****
reward (Reward)	**68.78%*****	**77.64%*****	**82.60%*****	**38.31%***	**54.60%*****	**53.18%****
risk (Risk)	**71.38%*****	**74.14%*****	**110.99%*****	49.94%	**61.38%*****	**78.82%*****

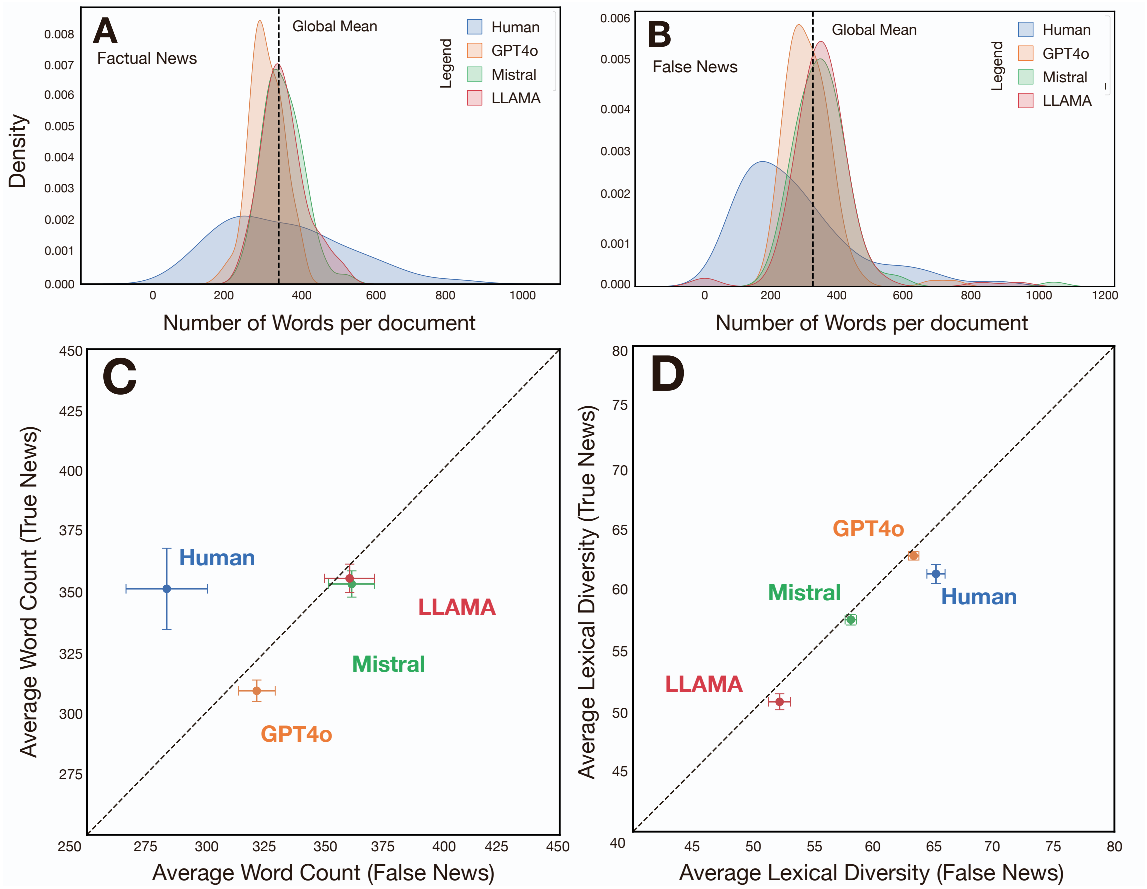

Figure S5: **Temperature: 0.7; Top$_p$: 0.8**, reproduction of Figure 1 with changed generation parameters. Descriptive comparison between the documents produced by Humans and LLMs. Panels A and B compare the distribution in terms of word count (length) of the articles with factual (A) and false (B) news. Panels C and D compare both factual and false news by source – Humans or LLMs – in terms of average document size (A) but also lexical diversity (D). Word Count (Factual/Fake News) and Lexical Diversity (Factual/Fake News) are represented as mean word count/lexical diversity ± standard deviation.

Table S10: **Temperature: 0.7; Top$_p$: 0.8**, reproduction of Table 2 with changed generation parameters. Salient Keywords Distinguishing Human-Authored and AI-Generated Texts Identified by SAGE. Expressions appearing twice between models are highlighted in bold.

Model	News Type	LLM Keywords	Human Keywords
**ChatGPT**	Factual News	**mas também** (-3.94); **além disso** (-3.49); não apenas (-3.41); **transparência** (-3.32); ainda mais (-3.23); recente (-3.13); à medida (-3.13); **enquanto isso** (-3.13); **especialmente** (-3.13); prometem (-3.02)	**novembro** (3.41); **vão** (3.34); **julho** (3.27); **referiu** (3.27); **vai** (3.21); **presidente da república** (3.19); **euros m2** (3.19); dezembro (3.13); **cinco** (3.11); duas (3.08)
**ChatGPT**	False News	**importância** (-4.30); **para garantir** (-3.74); **mas também** (-3.64); destacando (-3.04); **enquanto isso** (-2.95); gerou (-2.95); destacou (-2.85); críticas (-2.75); para evitar (-2.75); **incluindo** (-2.75)	**por isso** (4.14); **bastante** (3.65); **as pessoas** (3.51); idade (3.44); **são pessoas** (3.36); **km** (3.36); **dizer** (3.36); **vai** (3.33); **porque** (3.28); mil (3.17)
**Mistral**	Factual News	ministério da (-3.40); **transparência** (-3.40); adesão da suécia (-3.40); **especialmente** (-3.23); **mas também** (-3.23); para enfrentar (-3.23); **promover** (-3.03); esperança (-2.92); é crucial (-2.92); possam (-2.92)	face (3.54); **novembro** (3.48); conta (3.48); **porque** (3.42); **referiu** (3.34); florida (3.27); **presidente da república** (3.27); **euros m2** (3.27); **secretário-geral** (3.19); perante (3.19)
**Mistral**	False News	**importância** (-4.36); **para garantir** (-4.19); **ainda não** (-3.12); **incluindo** (-3.04); garantindo (-2.95); os consumidores (-2.95); do país (-2.86); é crucial (-2.86); **mas também** (-2.76); justiça (-2.76)	**por isso** (4.20); diz (3.82); **bastante** (3.70); **as pessoas** (3.57); **os seus** (3.49); **são pessoas** (3.41); **dizer** (3.41); **km** (3.41); quase (3.41); **vai** (3.38)
**Llama**	Factual News	equipe (-3.69); **além disso** (-3.69); vacinação contra a (-3.62); um dos principais (-3.55); sucesso (-3.38); inesquecível (-3.38); **promover** (-3.19); é um sinal (-3.08); se pronunciou (-3.08); **ainda não** (-3.08)	tendo (3.43); **novembro** (3.37); **porque** (3.31); **vão** (3.31); **julho** (3.24); **presidente da república** (3.16); **euros m2** (3.16); **secretário-geral** (3.08); **cinco** (3.08); quarta-feira (3.08)
**Llama**	False News	**promover** (-3.96); é fundamental (-3.83); um lembrete (-3.72); **importância** (-3.62); são conhecidos por (-3.43); **incluindo** (-3.43); pode ajudar (-3.29); dedo mindinho (-3.21); **enquanto isso** (-3.13); é importante (-3.13)	**por isso** (4.10); **porque** (3.64); **bastante** (3.60); **as pessoas** (3.47); quanto (3.47); **os seus** (3.39); algo (3.31); **km** (3.31); **dizer** (3.31); **são pessoas** (3.31)

Table S11: **Temperature: 0.7; Top$_p$: 0.8**, reproduction of Table 1 with changed generation parameters. Linguistic differences between human- and AI-generated texts across all five LIWC categories. Each cell shows the relative difference ($\Delta\%$) between each LLM (GPT-4o, Mistral, Llama) and the human baseline, along with the significance levels obtained using the Wilcoxon signed-rank test. Positive values indicate higher usage by LLMs; negative values indicate lower usage. Significance levels after Benjamini-Hochberg correction for multiple comparisons: $p < 0.05(^{*})$, $p < 0.01(^{**})$, $p < 0.001$ $(^{***})$.

**LIWC Category**	**Factual News**			**Fake News**		
	**ChatGPT**	**Mistral**	**Llama**	**ChatGPT**	**Mistral**	**Llama**
**Informal and Netspeak Attributes**						
informal (Informal Language)	**-62.65%****	**-65.37%*****	**-66.70%*****	**-48.57%*****	**-37.35%****	**-58.25%*****
netspeak (Netspeak)	-37.54%	**-81.94%***	**-71.52%***	**-80.07%****	**-66.65%****	**-90.67%****
**Emotional and Affective Attributes**						
affect (Affect)	**87.66%*****	**95.96%*****	**121.27%*****	**50.39%*****	**49.94%****	**65.82%*****
posemo (Positive Emotions)	**98.11%*****	**110.67%*****	**132.98%*****	**60.56%****	**75.57%****	**80.21%*****
negemo (Negative Emotions)	26.07%	23.34%	34.08%	18.10%	0.67%	29.82%
anx (Anx)	15.32%	-12.59%	10.19%	**-46.84%****	**-46.36%****	-28.02%
anger (Anger)	**-22.75%***	-18.89%	-18.89%	**-32.88%****	**-43.02%****	-21.19%
sad (Sad)	**-39.23%****	**-52.97%*****	-18.64%	-7.29%	**-33.55%****	36.17%
**Cognitive Attributes**						
cogproc (Cognitive Processes)	**49.38%*****	**52.15%*****	**63.73%*****	20.73%	26.14%	45.00%
insight (Insight)	**56.04%***	32.26%	28.59%	**44.87%***	45.62%	49.77%
cause (Causal)	**54.55%*****	**58.58%*****	**76.06%*****	**43.80%***	38.49%	**40.13%***
discrep (Discrepancies)	0.02%	16.61%	**40.07%*****	**-4.11%***	**-2.10%***	10.58%
tentat (Tentative)	47.22%	**65.58%***	**93.28%*****	**-6.03%***	**1.34%***	22.99%
certain (Certainty)	**134.86%*****	**137.91%*****	**106.35%*****	26.26%	38.02%	10.12%
differ (Differentiation)	23.14%	23.28%	27.62%	-1.46%	9.46%	42.91%
**Perceptual Attributes**						
percept (Perceptual Processes)	29.87%	**60.47%****	38.52%	-1.67%	-1.91%	6.84%
see (See)	32.44%	48.47%	26.51%	22.46%	42.36%	20.00%
hear (Hear)	**-51.57%*****	**-32.64%*****	**-29.42%****	**-70.98%*****	**-32.66%****	**-34.94%****
feel (Feel)	-0.04%	-1.89%	**-15.50%*****	**-26.35%****	**-38.61%*****	**-19.52%***
**Motivational and Drive Attributes**						
drives (Drives)	**41.94%*****	**42.32%*****	**53.74%*****	**36.47%*****	**32.99%*****	**52.88%*****
affiliation (Affiliation)	**66.71%****	**49.03%***	52.21%	9.07%	11.22%	27.59%
achieve (Achievement)	**111.64%*****	**89.62%*****	**122.29%*****	**54.87%*****	**64.63%*****	**91.40%*****
power (Power)	19.56%	26.27%	21.28%	**64.24%*****	**47.28%****	**43.76%****
reward (Reward)	**72.39%*****	**73.65%*****	**82.27%*****	**41.42%***	**42.82%***	**56.33%***
risk (Risk)	**82.09%*****	**84.71%*****	**107.97%*****	**50.26%****	**50.00%***	**70.65%*****

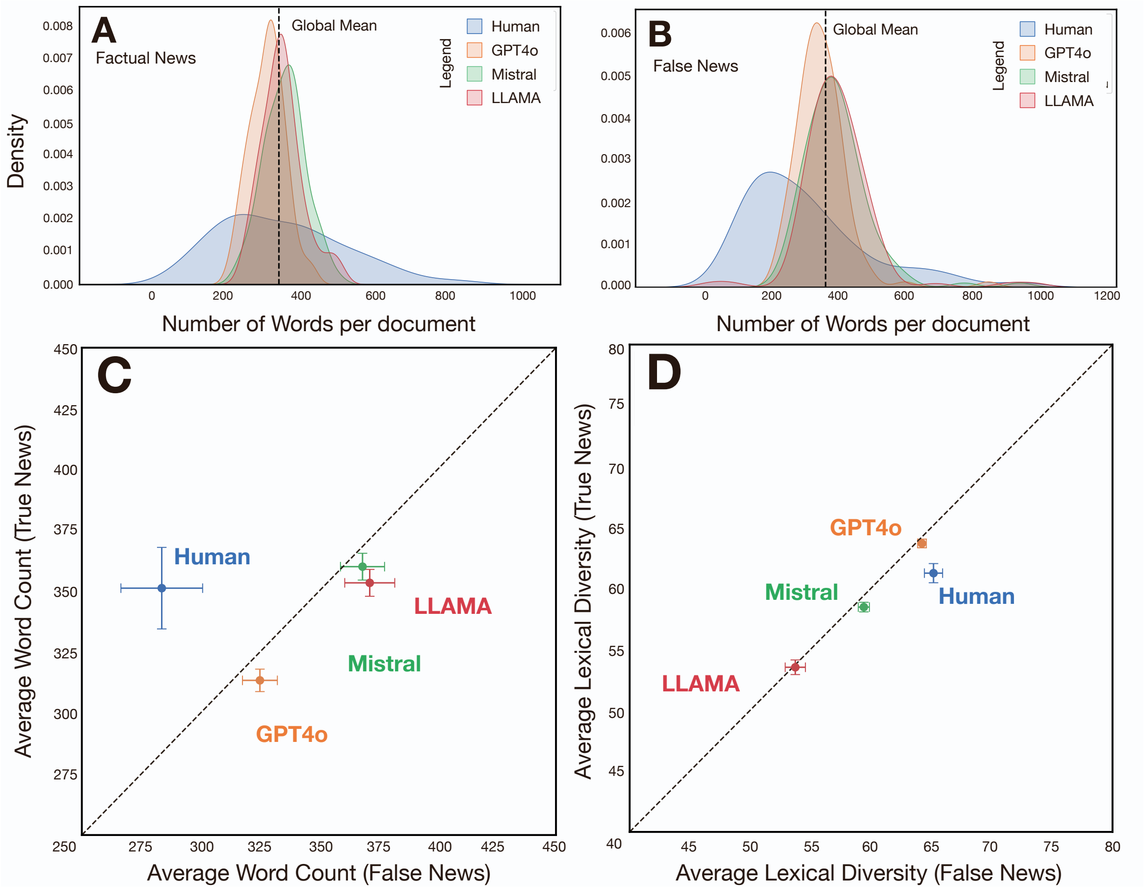

Figure S6: **Temperature: 0.9; Top$_p$: 1**, reproduction of Figure 1 with changed generation parameters. Descriptive comparison between the documents produced by Humans and LLMs. Panels A and B compare the distribution in terms of word count (length) of the articles with factual (A) and false (B) news. Panels C and D compare both factual and false news by source – Humans or LLMs – in terms of average document size (A) but also lexical diversity (D). Word Count (Factual/Fake News) and Lexical Diversity (Factual/Fake News) are represented as mean word count/lexical diversity ± standard deviation.

Table S12: **Temperature: 0.9; Top$_p$: 1**, reproduction of Table 2 with changed generation parameters. Salient Keywords Distinguishing Human-Authored and AI-Generated Texts Identified by SAGE. Expressions appearing twice between models are highlighted in bold.

**Model**	**News Type**	**LLM Keywords**	**Human Keywords**
**ChatGPT**	Factual News	**mas também** (-4.03); reforçar (-3.43); determinação (-3.35); recente (-3.17); significativas (-3.06); especialmente (-3.06); enfrenta (-2.95); **não apenas** (-2.95); à medida (-2.95); um ambiente (-2.95)	**novembro** (3.46); quase (3.46); **referiu** (3.32); **presidente da república** (3.25); **florida** (3.25); **euros m2** (3.25); família (3.17); **secretário-geral** (3.17); cinco (3.17); duas (3.14)
**ChatGPT**	False News	**importância** (-4.05); **mas também** (-3.86); **não só** (-3.17); **gestão** (-3.10); especialistas em (-3.02); **políticas** (-2.93); proteção (-2.84); para evitar (-2.84); promover (-2.74); ao longo (-2.74)	**por isso** (4.20); **vai** (4.08); vão (3.57); **as pessoas** (3.57); **os seus** (3.50); **são pessoas** (3.42); **km** (3.42); dizer (3.42); porque (3.34); **radar** (3.23)
**Mistral**	Factual News	**mas também** (-3.48); para enfrentar (-3.33); transparência (-3.33); possam (-3.16); uma série (-3.16); adesão da suécia (-3.16); ministério da (-3.07); para mais informações (-2.97); especialmente (-2.86); tensão (-2.86)	conta (3.51); **referiu** (3.38); **vai** (3.31); **presidente da república** (3.30); **euros m2** (3.30); cinco (3.22); perante (3.22); **secretário-geral** (3.22); suécia à nato (3.13); esta terça-feira (3.13)
**Mistral**	False News	para garantir (-4.25); **importância** (-3.55); importância da (-2.97); perto (-2.97); polémica (-2.97); **políticas** (-2.89); **mas também** (-2.80); **justiça** (-2.80); é crucial (-2.80); para evitar (-2.80)	**por isso** (4.23); bastante (3.74); **as pessoas** (3.60); logo (3.60); depósito (3.60); **os seus** (3.52); **são pessoas** (3.44); **km** (3.44); pessoa (3.35); **radar** (3.25)
**Llama**	Factual News	além disso (-4.30); marcelo rebelo (-3.70); **mas também** (-3.70); equipe (-3.64); **não apenas** (-3.57); promover (-3.33); transparência (-3.33); um desafio (-3.23); sucesso (-3.23); oferece uma (-3.13)	duas (3.78); marcelo rebelo de (3.68); comunicado (3.58); quinta-feira (3.53); donald trump (3.41); quase (3.41); **novembro** (3.41); **referiu** (3.27); **florida** (3.19); **euros m2** (3.19)
**Llama**	False News	um lembrete (-3.97); incluindo (-3.84); **importância** (-3.84); promover (-3.48); é fundamental (-3.48); **políticas** (-3.35); **gestão** (-3.35); **mas também** (-3.21); **não apenas** (-2.86); **justiça** (-2.86)	**por isso** (4.15); diz (3.78); **as pessoas** (3.52); **os seus** (3.45); **são pessoas** (3.36); **km** (3.36); **radar** (3.17); av (3.17); próprio (3.06); cabeça (3.06)

Table S13: **Temperature: 0.9; Top$_p$: 1**, reproduction of Table 1 with changed generation parameters. Linguistic differences between human- and AI-generated texts across all five LIWC categories. Each cell shows the relative difference (Δ%) between each LLM (GPT-4o, Mistral, Llama) and the human baseline, along with the significance levels obtained using the Wilcoxon signed-rank test. Positive values indicate higher usage by LLMs; negative values indicate lower usage. Significance levels after Benjamini-Hochberg correction for multiple comparisons: $p < 0.05(^{*})$, $p < 0.01(^{**})$, $p < 0.001$ $(^{***})$.

**LIWC Category**	**Factual News**			**Fake News**		
	**ChatGPT**	**Mistral**	**Llama**	**ChatGPT**	**Mistral**	**Llama**
**Informal and Netspeak Attributes**						
informal (Informal Language)	**-66.56%*****	**-66.77%*****	**-71.29%*****	**-66.05%*****	**-60.18%*****	**-60.17%*****
netspeak (Netspeak)	**-81.79%***	**-81.22%***	**-82.00%***	**-85.55%*****	**76.77%****	**-72.36%***
**Emotional and Affective Attributes**						
affect (Affect)	**99.19%*****	**93.99%*****	**106.73%*****	**52.43%*****	**46.24%****	**71.38%*****
posemo (Positive Emotions)	**102.76%*****	**106.18%*****	**118.07%*****	**66.49%****	**62.71%****	**79.50%*****
negemo (Negative Emotions)	28.34%	18.14%	**41.12%***	11.16%	16.90%	16.45%
anx (Anx)	4.70%	-16.14%	27.68%	**-44.43%****	**-42.42%****	-3.54%
anger (Anger)	**-27.63%****	**-26.85%****	-13.53%	**-40.56%*****	**-33.57%***	**-39.43%****
sad (Sad)	**-37.37%****	**-35.76%***	**-29.98%***	**-19.67%***	**-24.30%***	-2.52%
**Cognitive Attributes**						
cogproc (Cognitive Processes)	**51.85%*****	**50.56%*****	**59.55%*****	22.60%	26.90%	**47.49%****
insight (Insight)	**50.23%****	33.74%	**73.44%***	**61.80%***	37.81%	**63.91%***
cause (Causal)	**60.79%*****	**62.29%*****	**80.52%*****	**37.91%****	**38.24%***	**45.88%****
discrep (Discrepancies)	17.91%	16.18%	23.82%	**-11.36%*****	**-6.93%*****	10.37%
tentat (Tentative)	56.00%	35.30%	**69.51%***	**-9.47%****	6.47%	20.36%
certain (Certainty)	**113.95%*****	**128.17%*****	**104.96%*****	**45.55%***	**36.96%***	40.79%
differ (Differentiation)	35.09%	20.54%	25.87%	**3.43%****	10.49%	37.96%
**Perceptual Attributes**						
percept (Perceptual Processes)	31.42%	54.46%	**50.76%***	-0.53%	7.08%	11.55%
see (See)	**63.40%***	35.73%	73.13%	35.88%	36.47%	31.76%
hear (Hear)	**-58.68%*****	**-29.48%****	**-35.34%*****	**-60.52%*****	**-47.83%*****	**-25.15%***
feel (Feel)	-2.00%	-1.25%	4.51%	**-15.15%***	**-27.72%*****	**-13.36%***
**Motivational and Drive Attributes**						
drives (Drives)	**45.15%*****	**42.77%*****	**56.89%*****	**35.10%*****	**35.09%*****	**49.04%*****
affiliation (Affiliation)	**72.88%*****	**43.58%***	51.58%	8.41%	17.50%	18.90%
achieve (Achievement)	**106.45%*****	**99.83%*****	**134.28%*****	**66.11%*****	**65.11%*****	**83.83%*****
power (Power)	20.39%	21.38%	**38.87%****	**67.54%*****	**47.81%*****	**64.93%*****
reward (Reward)	**70.03%*****	**74.43%*****	**94.08%*****	**41.73%***	**49.26%****	**49.42%****
risk (Risk)	**85.54%*****	**72.60%*****	**107.47%*****	**43.93%***	**58.68%****	**67.03%*****
